# Modeling of Deformable Objects for Robotic Manipulation: A Tutorial and Review

**DOI:** 10.3389/frobt.2020.00082

**Published:** 2020-09-17

**Authors:** Veronica E. Arriola-Rios, Puren Guler, Fanny Ficuciello, Danica Kragic, Bruno Siciliano, Jeremy L. Wyatt

**Affiliations:** ^1^Department of Mathematics, Faculty of Science, UNAM Universidad Nacional Autonoma de Mexico, Ciudad de México, Mexico; ^2^Autonomous Mobile Manipulation Laboratory, Centre for Applied Autonomous Sensor Systems, Orebro University, Orebro, Sweden; ^3^PRISMA Laboratory, Department of Electrical Engineering and Information Technology, University of Naples Federico II, Naples, Italy; ^4^Robotics, Learning and Perception Laboratory, Centre for Autonomous Systems, EECS, KTH Royal Institute of Technology, Stockholm, Sweden; ^5^School of Computer Science, University of Birmingham, Birmingham, United Kingdom

**Keywords:** deformable objects, shape representation, learning of deformation, control of deformable objects, registration of shape deformation, tracking of deformation

## Abstract

Manipulation of deformable objects has given rise to an important set of open problems in the field of robotics. Application areas include robotic surgery, household robotics, manufacturing, logistics, and agriculture, to name a few. Related research problems span modeling and estimation of an object's shape, estimation of an object's material properties, such as elasticity and plasticity, object tracking and state estimation during manipulation, and manipulation planning and control. In this survey article, we start by providing a tutorial on foundational aspects of models of shape and shape dynamics. We then use this as the basis for a review of existing work on learning and estimation of these models and on motion planning and control to achieve desired deformations. We also discuss potential future lines of work.

## 1. Introduction

Robotic manipulation work tends to focus on rigid objects (Bohg et al., 2014; Billard and Kragic, 2019). However, most objects manipulated by animals and humans change shape upon contact. Manipulating a deformable object presents a quite different set of challenges from those that arise when manipulating a rigid object. For example, forces applied to a rigid object simply sum to determine the external wrench and, when integrated over time, result in a sequence of rigid body transformations in SE(3). This is relatively simple dynamic model, albeit still difficult to estimate for a given object, manipulator, and set of environment contacts.

Forces applied to a deformable body, by contrast, both move the object and change its *shape*. The exact combination of deformation and motion depends on the precise material composition. Thus, *material properties* become a critical part of the system dynamics, and consequently the underlying physics of deformation is complex and hard to capture. In addition, the *dynamics* models typically employed in high-fidelity mechanical modeling—such as finite element models—while precise, require detailed knowledge of the material properties, which would be unavailable to a robot in the wild. Yet such a lack of detailed physics knowledge does not prevent humans and other animals from performing dexterous manipulation of deformable objects. Consider the way a New Caledonian crow shapes a tool from a branch (Weir and Kacelnik, 2006) or how a pizzaiolo dexterously transforms a ball of dough into a pizza. Clearly, robots have a long way to go to match these abilities.

Even though a great deal of work has been done on developing solutions for each of these stages (Cootes et al., 1992; Montagnat et al., 2001; Nealen et al., 2006; Moore and Molloy, 2007), only a few combinations have actually been tried in robotics to date (Nadon et al., 2018; Sanchez et al., 2018). In contrast to earlier review articles, this paper consists of both a tutorial and a review of related work, aimed at newcomers to robotic modeling and manipulation of deformable objects who need a quick introduction to the basic methods, which are adopted from various other fields and backgrounds. We provide, in a single place, a menu of possible approaches motivated by computer vision, computer graphics, physics, machine learning, and other fields, in the hope that readers can find new material and inspiration for creatively developing their work. We then review how these methods are applied in practice. In this way, we take a more holistic approach than existing reviews. Since the literature in the fields we touch on is abundant, we cannot be exhaustive and will focus mostly on manipulation of volumetric solid objects.

**Figure 1:**
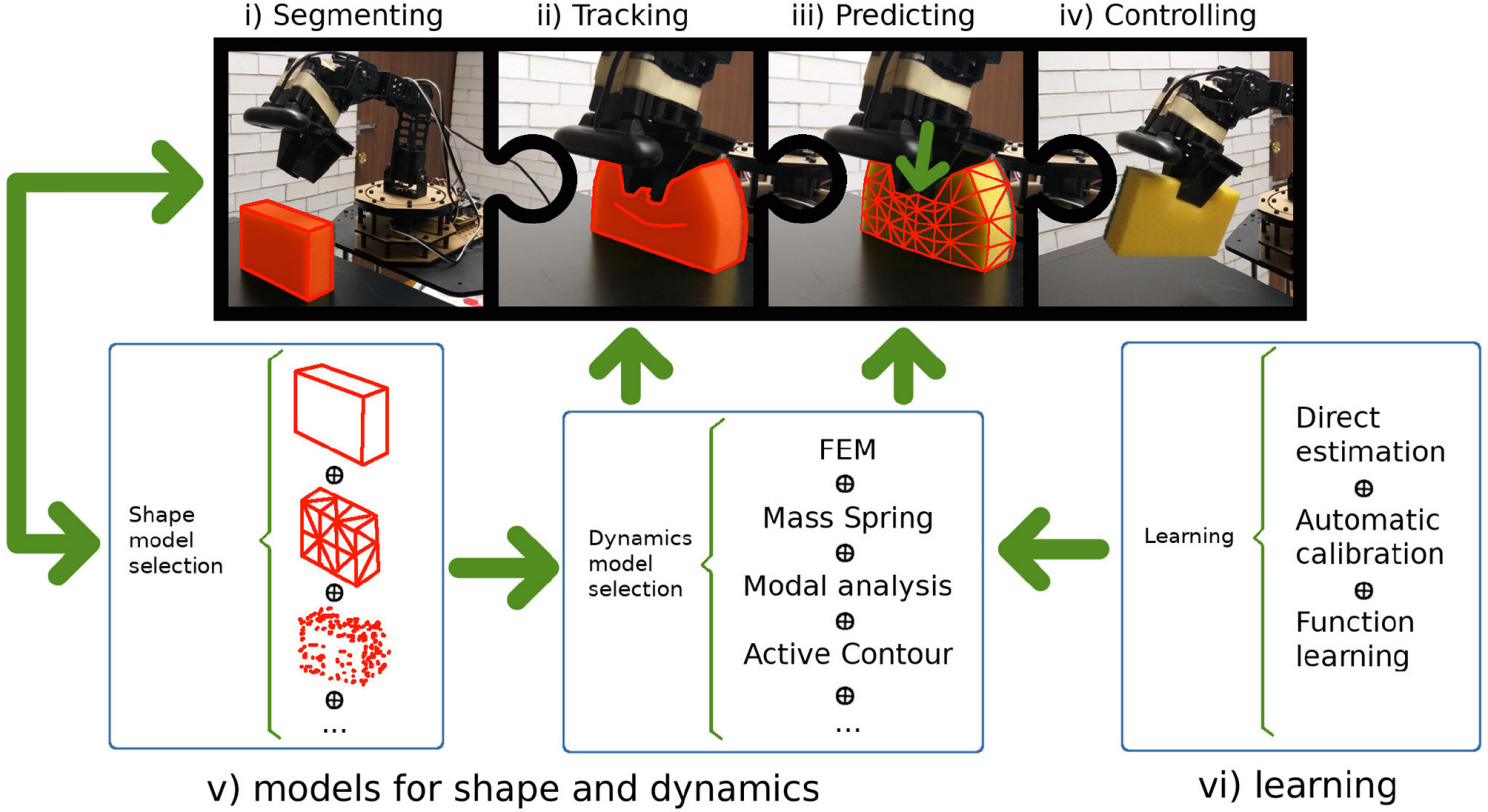
A general purpose robot must be capable of: **(i)** perceiving and segmenting the object from the scene; **(ii)** tracking the object's motion; **(iii)** predicting the object's behavior; **(iv)** planning and controlling new manipulation strategies based on the predictions; **(v)** selecting the appropriate model for each task, since all models for shape and dynamics have limitations; and **(vi)** learning new models for previously unknown shapes and materials.

This review is motivated by a future goal in the form of an ideal scenario. In this scenario a general purpose robot would be capable of perceiving *shape, dynamics*, and necessary *material properties* (e.g., elasticity, plasticity) of deformable objects to implement manipulation strategies relying on planning and control methods. Currently no robot has all these capabilities. Therefore, we decompose the problem space into five main parts that work like pieces in a puzzle: Because representational choices are fundamental, we explain, in a tutorial style, (1) the modeling of shape (section 2) and (2) the modeling of deformation dynamics (section 3) to provide the reader with the necessary mathematical background; then we discuss, in survey form, (3) learning and estimation of the parameters of these models that are related to deformability of objects (e.g., material properties, such as elasticity, or shape properties, such as resolution of a mesh; section 4), (4) the application of the models to perception and prediction, and (5) planning and control of manipulation actions (section 5), since these topics build on the models explained in (1) and (2) and there is such a wide range of different approaches that it would be impossible to cover them all in depth. Figure 1 shows our guideline processing stream: (i) The robot perceives the object, segments it from its environment, and selects an adequate representation for its shape and intended task; the desired type of representation will determine which algorithms must be used to recognize the object. (ii) As the robot interacts with the object, it deforms the object and must register the deformation by modifying the shape representation accordingly; while doing so, it can make use of tracking/registration techniques or enhanced predictive tracking (which requires a model of the dynamics). (iii) A suitable model for the dynamics is selected to predict new configurations of the shape representation as the robot interacts with the object. (iv) Information from the previous stages is used to integrate the inputs and rules for the control strategies. Finally, all this information can be fed into learning algorithms capable of increasing the repertoire of known objects. At this stage, there are three types of strategies: (a) estimating new parameters directly (which is rarely viable); (b) calibrating known physics-based models automatically by allowing the robot to take some measurements that will help to determine the value of model parameters; and (c) approximating new functions that describe the dynamics, as is done with neural networks.

**Figure 2:**
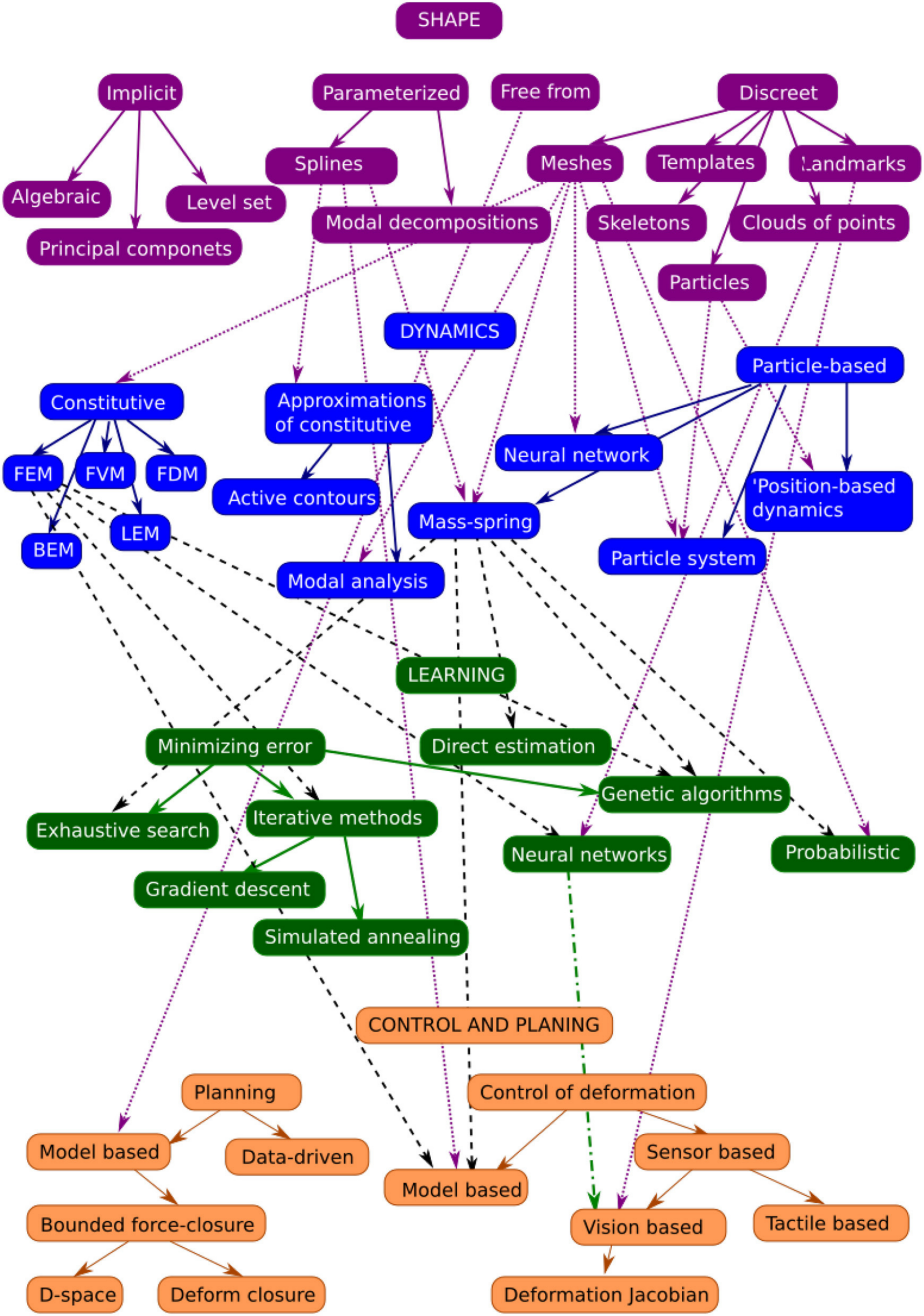
Relationships between shape, dynamics, learning models, and control methodologies as they have been used in the literature. Colored arrows indicate subcategories, while black arrows show when a methodology in one level has been used for the next one.

Figure 2 shows the connections between the different models covered in this review as they have been used in the publications mentioned. Table 1 gives a summary of the publications discussed in each section. The following describes the notation that will be used throughout the paper:

- Scalars: italic lower-case letters, e.g., *x*, *y*, *z*.
- Vectors: bold lower-case letters, e.g., **p** = {*x, y, z*}^*T*^.
- Matrices: bold upper-case letters, e.g., **R**.
- Scalar functions whose range is ℝ: italic lower-case letters followed by parentheses, e.g., *f*(·).
- Vector functions whose range is ℝ^*n*^ with *n* > 1: bold italic letters followed by parentheses, e.g., ***S***(·).
- Sets: calligraphic letters, e.g., S.
- Number sets: blackboard bold upper-case letters, e.g., ℝ, ℕ.

**Table 1:** Publication summary based on some papers from each section.

Shape	Implicit	Algebraic	Gascuel, 1993; Kumar et al., 1995; Jaklic et al., 2000
		Level set	Sethian, 1997; Cremers, 2006; Sun et al., 2008
		Eigenmodes	Cootes et al., 1995; Blake et al., 1998; Leventon et al., 2000; Tsai et al., 2001; Cremers, 2006
	Parameterized	Splines	de Boor, 1976; Catmull and Clack, 1978; Kass et al., 1988; Gibson and Mirtich, 1997; Unser, 1999; Cordero Valle and Cortes Parejo, 2003; Sederberg et al., 2003; Maraffi, 2004; Song and Bai, 2008; Prasad et al., 2010
		Modal decomposition	Szekely et al., 1995
	Free-form		Sederberg and Parry, 1986; Moore and Molloy, 2007
		Multigrid	Xian et al., 2019
	Discrete	Meshes	Delingette, 1999; Montagnat et al., 2001; Arvanitis et al., 2019
		Skeletons	Schaefer and Yuksel, 2007
		Templates	Yuille et al., 1992; Basri et al., 1998; Ravishankar et al., 2008; Arriola-Rios et al., 2013; Gallardo et al., 2020
		Landmarks	Blake et al., 1998; Cootes and Taylor, 2004
		Particles	Nealen et al., 2006
		Cloud of points	Cretu et al., 2009; Newcombe and Davison, 2010; Martínez et al., 2019; Makovetskii et al., 2020
Dynamics	Particle-based	Particle systems	Tonnesen and Terzopoulos, 2000
		Mass-spring systems	Bianchi et al., 2004; Teschner et al., 2004; Morris and Salisbury, 2008; Schulman et al., 2013; Arriola-Rios and Wyatt, 2017
		Neural networks	Nurnberger et al., 1998; Zhang et al., 2019
		Position-based	Müller et al., 2005; Zhu et al., 2008; Tian et al., 2013; Macklin et al., 2014; Sidorov and Marshall, 2014; Guler et al., 2017; Romeo et al., 2020
	Constitutive	FEM	Essa et al., 1992; Frank et al., 2014; Petit et al., 2018
		FVM	Teran et al., 2003; Barth et al., 2018
		FDM	Terzopoulos et al., 1987
		BEM	Greminger and Nelson, 2008
		LEM	Balaniuk and Salisbury, 2002
	Approximations	Modal analysis	Pentland and Williams, 1989; Barbič and James, 2005; Fulton et al., 2019
		Active contours	Kass et al., 1988; Ahlberg, 1996; Nisirat, 2019
Learning	Discrete		Gelder, 1998
	Minimizing error	Exhaustive search	Guler et al., 2015
		Iterative methods	Teschner et al., 2004; Frank et al., 2014
		Genetic algorithms	Bianchi et al., 2004
		Neural networks	Cretu et al., 2012
	Probability		Risholm et al., 2010; Schulman et al., 2013
Control and planning	Planning	Model-based	Gopalakrishnan and Goldberg, 2004; Das and Sarkar, 2011; Frank et al., 2014
		Data-driven	Mira et al., 2015; Li et al., 2016
	Control	Model-based	Largilliere et al., 2015; Lin et al., 2015; Zaidi et al., 2017; Ficuciello et al., 2018
		Sensor-based	Wada et al., 2001; Smolen and Patriciu, 2009; Berenson, 2013; Navarro-Alarcon et al., 2016; Delgado et al., 2017b; Hu et al., 2019; Cherubini et al., 2020

## 2. Representing Shape for Deformable Objects

The initial problem of manipulating a deformable object is to perceive and segment the object shape from the scene as it deforms (Figure 1i). The main difficulty with this problem is the number of degrees of freedom required to model the object shape. The expressiveness, accuracy, and flexibility of the model can ease the modeling of the dynamics or make it more difficult in different scenarios. For this reason, this section introduces a variety of models from mathematics, computer graphics, and computer vision for representation of the shape of deformable objects.

### 2.1. Implicit Curves and Surfaces

**Figure 3:**
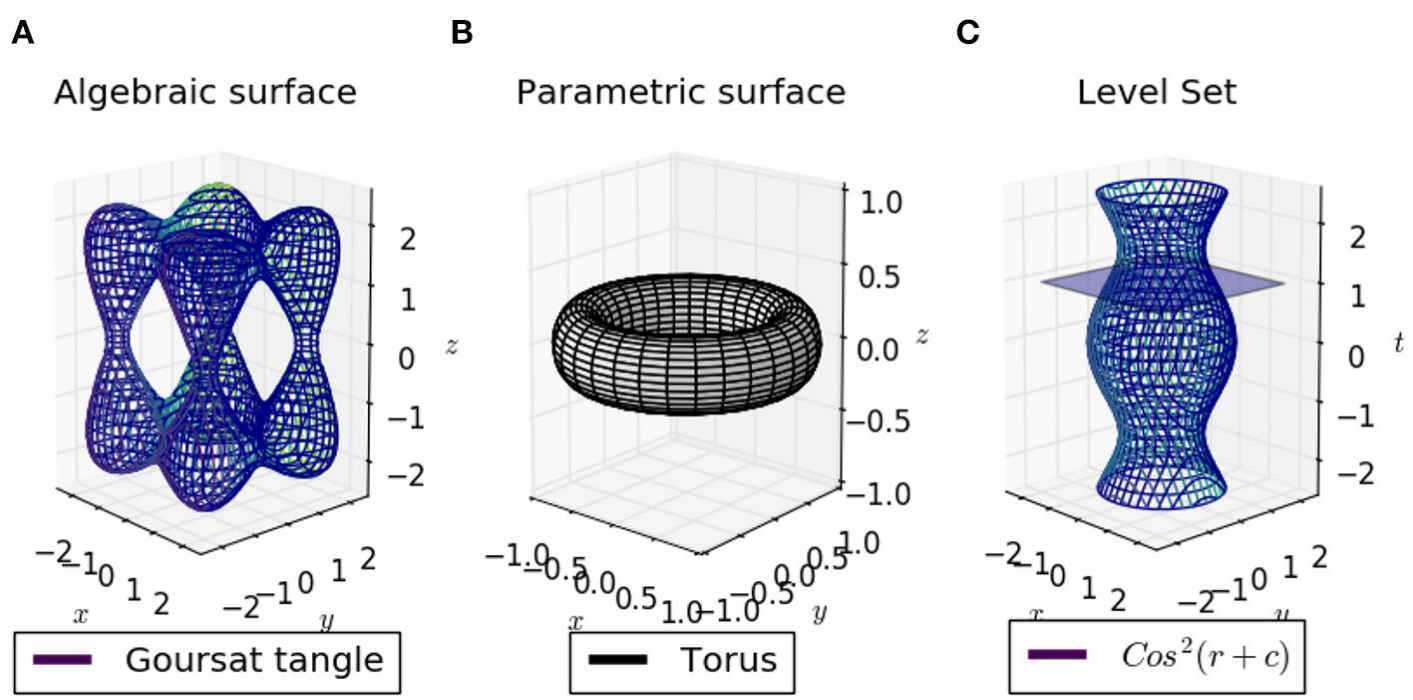
**(A)** Algebraic surfaces can represent basic and complex shapes, such as circles or the tangled cube; a limited but useful set of deformations is straightforward to define, while other deformations that are not so intuitive tend to be used in combination with skeletons and skinning techniques. **(B)** Parametric surfaces can represent a wider variety of shapes and their great flexibility allows deformation to be controlled with more intuitive parameters; therefore their use in connection with models of dynamics is considerable. **(C)** Level set curves can show the evolution of a 2D shape in time; the intersection of a time plane with the surface in 3D is a level curve, which represents the 2D contour shape of the object at a given time *t*. In this example cos^2^(*r* + *c*) is the mathematical representation of the 2D contour shape's evolution through time.

An implicit curve or surface of dimension *n* − 1 is generally defined as the zero set of a function *f*:ℝ^*n*^ → ℝ,

(1) $$\begin{array}{l} S_{f}=\left\{\text{p}\in {\mathbb{R}}^{n}|f\left(\text{p}\right)=0\right\}, \end{array}$$

where **p** is a coordinate in an *n*-dimensional space in which the surface is embedded (Montagnat et al., 2001). Therefore, the set $S_{f}$ defines the surface formed by all points **p** in ℝ^*n*^ such that the function *f*, when evaluated at **p**, is equal to zero. The implicit function is used to locate surface points by solving the equation *f* (**p**) = 0 (Figure 3A). In robotics, *n* is usually 3 to represent Cartesian coordinates, but sometimes an extra dimension can be used to represent time. Representations that fall within this category are explained in the rest of this subsection.

#### 2.1.1. Algebraic Curves and Surfaces

Algebraic curves and surfaces satisfy (1) with *f* (**p**) being a polynomial. First-degree polynomials define *planes* and *hyperplanes*; second-degree polynomials define *conics*, which include circles, ellipses, parabolas, and hyperbolas, and *quadrics*, which include ellipsoids, paraboloids, hyperboloids, toroids, cones, and cylinders; their (*n* − 1)-dimensional extensions are surfaces in an *n*-dimensional space that satisfy the equation

(2) $$\begin{array}{l} f\left(\text{p}\right)={\text{p}}^{T}\text{A}\text{p}+\text{b}\text{p}+c=0 \end{array}$$

where $\text{p}={\left\{x_{1},x_{2},\ldots ,x_{n}\right\}}^{T}\in {\mathbb{R}}^{n}$ is a column vector, **p**^*T*^ denotes the transpose of **p** (a row vector), **A** ∈ ℝ^*n*×*n*^ is a matrix, **b** ∈ ℝ^*n*^ is a row vector, and *c* is a scalar constant. Note that all the aforementioned shapes are included as particular cases of this definition. To define a particular shape, which satisfies a given set of constraints, the constant values in **A**, **b**, and *c* must be determined; for example, with *n* = 2, the constants that define a circle passing through a given set of three points can be found by solving the system of three equations where *f* (**p**_*i*_) = 0 for all *i* and *f* (**p**_*i*_) is a second-degree polynomial. In some contexts the same equation can be rewritten to facilitate this estimation; for example, it is easy to determine the circle centered at (*x*_*c*_, *y*_*c*_) with radius *r* if the second-degree polynomial is written as ${\left(x-x_{c}\right)}^{2}+{\left(y-y_{c}\right)}^{2}=r^{2}$ with **p** = {*x, y*}.

*Superquadrics* are defined by second-degree polynomials, while *hyperquadrics* are the most general form and allow the representation of complex non-symmetric shapes; they are given by the equation

(3) $$\begin{array}{l} \overset{N}{\underset{i\;=\;1}{\sum }}|a_{i}x+b_{i}y+c_{i}z+d_{i}{|}^{\gamma_{i}}=1 \end{array}$$

where **p** = {*x, y, z*}^*T*^ ∈ ℝ^3^, *N* is an arbitrary number of planes whose intersection surrounds the object, *a, b, c*, and *d* are shape parameters of these planes, and γ_*i*_ ∈ ℝ with γ_*i*_ ≥ 0 for all *i*. Kumar et al. (1995) presented a method for fitting hyperquadrics to very complex deformable shapes registered as range data. A model like (3) could be used as the base representation for a model of the dynamics if the deformations are small. It can be used in combination with local surface deformations to represent a wider set of surfaces. For example, if the superquadric is the set of points Q that satisfy the corresponding equation, a deformed model could be given by S=c+R(Q+d) where **c** represents the inertial center of the superquadric, **R** is a rotation matrix, and **d** is a vectorial displacement field. Other types of deformation can be defined as well (Montagnat et al., 2001). For more information about superquadrics, see Jaklic et al. (2000).

Algebraic curves, surfaces, and volumes can be used as 1D, 2D, and 3D skeletons, or to represent objects of similar shapes and deformations (Gascuel, 1993). They are easy to deform in certain cases, but the types of deformations that are straightforward to apply are very limited, such as matrix transformations that bend, pitch, twist, stretch, or translate all the points on a curve or the space in which the curve is embedded. For this reason, algebraic curves and surfaces are more suited to representing articulated or semi-articulated objects. Objects can also be composed of several algebraic curves, where each component is easy to manipulate with this representation. To model more complex deformed shapes, Raposo and Gomes (2019) introduced products of primitive algebraic surfaces, such as spheres and cylinders, which enable both local and global deformations to be controlled more easily than in traditional algebraic shape models. Moreover, these models can be combined with skinning techniques to emulate soft deformable objects and also have parameterized representations.

#### 2.1.2. Level Set Methods

In level set methods, the deformable model is embedded in a higher-dimensional space, where the extra dimension represents time (Sethian, 1997; Montagnat et al., 2001). A hypersurface **Ψ** is defined by **Ψ**(**p**, 0) = dist(**p**, ***S***_0_), where ***S***_0_ is the initial surface and dist can be the signed Euclidean distance between a point **p** and the surface. The distance is positive if the point lies outside the surface and negative otherwise. The evolution of the surface ***S*** is governed by the partial differential equation **Ψ**_*t*_ + |∇**Ψ**|*F* = 0 involving the function **Ψ**(**p**, *t*) and a speed function *F*, which determines the speed at which each point of the surface must be moved. Thus, the function **Ψ** evolves in time and the current shape corresponds to the surface given by **Ψ**(**p**, *t*) = 0 (Figure 3C). The function **Ψ** could have any form, such as a parameterized one, **Ψ** = **Ψ**(*x* (*u, v*), *y* (*u, v*), *z* (*u, v*), *t*), with the surface still defined in implicit form, **Ψ**(*x* (*u, v*), *y* (*u, v*), *z* (*u, v*), *t*) = **0**. Unfortunately, it could also be that **Ψ** does not even have an algebraic expression and may need to be approximated with numerical methods. The main advantage of level set methods is that they allow changes of surface topology implicitly. The set S may split into several connected components, or several distinct components may merge, while **Ψ** remains a function.

In computer vision applications, such as human tracking and medical imaging, level set methods have been used successfully in tracking deformable objects (Sethian, 1997; Cremers, 2006). For example, Sun et al. (2008) recursively segmented deformable objects across a sequence of frames using a low-dimensional modal representation and applied their technique to left ventricular segmentation across a cardiac cycle. The dynamics are represented using a distance level set function, whose representation is simplified using *principal component analysis* (PCA). Sun et al. used methods of particle-based smoothing as well as non-parametric belief propagation on a loopy graphical model capturing the temporal periodicity of the heart, with the objective being to estimate the current state of the object not only from the data observed at that instant but also from predictions based on past and future boundary estimates. Even though we did not find examples of this method being used in robotics, it seems a suitable candidate since it models the shape change through time implicitly and would thus allow the robot to keep track of the evolving shape of an object during manipulation.

#### 2.1.3. Gaussian Principal Component Eigenmodes

This kind of representation is valid when the types of deformations can be described with a single mathematical formulation. Given a representative set $S_{N}=\left\{S_{0},S_{1},\ldots ,S_{N-1}\right\}$ of the types of surface deformation that objects can undergo, it is possible to use PCA to detect the main modes of deformation (i.e., the eigenmodes **Φ**_*n*_) and thus re-express the shapes as a linear combination of those modes. Hence a new shape estimation can be done using

(4) $$\begin{array}{l} \bar{S}=S_{\mu }+\alpha \Phi_{n} \end{array}$$

where **Φ**_*n*_ (*n* ≪ *N*) is the largest eigenmode of shape variations in ***S***_*N*_, ***S***_μ_ is the mean of the representative set of shapes ***S***_*N*_, and α is a set of coefficients. Such an eigenmode representation is useful for dealing with missing or misleading information (e.g., noise or occlusions) coming from sensory data while constructing the shape of the object (Cootes et al., 1995; Blake et al., 1998; Cremers, 2006; Sinha et al., 2019).

Employing combinations of previously cited methods, Leventon et al. (2000) used eigenmode representation with level set curves to segment images, such as medical images of the femur and corpus callosum, by defining a probability distribution over the variances of a set of training shapes. The segmentation process embeds an initial curve as the zero level set of a higher-dimensional surface, and then evolves the surface such that the zero level set converges on the boundary of the object to be segmented. At each step of the surface evolution, the maximum *a posteriori* position and shape of the object in the image were estimated based on the prior shape information and the image information. The surface was then evolved globally toward the maximum *a posteriori* estimate and locally based on image gradients and curvature. The results were demonstrated on synthetic data and medical images, in both 2D and 3D. Tsai et al. (2001) further developed this idea.

### 2.2. Explicit Parameterized Representations

Explicit parameterized representations are evaluated directly from their functional definition (Figure 3B). In 3D, they are of the form ***C***(*u*) = {*x* (*u*), *y* (*u*), *z* (*u*)}^*T*^ for curves and ***S***(*u, v*) = {*x* (*u, v*), *y* (*u, v*), *z* (*u, v*)}^*T*^ for surfaces, where *u* and *v* are parameters. The curve or surface is traced out as the values of the parameters are varied. For example, if *u* ∈ [0, 1], the shape is traced out as *u* varies from 0 to 1, as happens with the circle

(5) $$\begin{array}{l} C\left(u\right)=\left\{\begin{array}{c} x=cos\left(u\right)\\ y=sin\left(u\right) \end{array}\right\}. \end{array}$$

It is common practice to parameterize a curve by time *t* if it will represent a trajectory, or by arc length *l*^1^.

#### 2.2.1. Splines

A mathematical spline ***S*** is a piecewise-defined real function, with *k* polynomial pieces *s*_*i*_(*u*) parameterized by *u* ∈ [*u*_0_, *u*_*k*_], used to represent curves or surfaces (Cordero Valle and Cortes Parejo, 2003). The order *n* of the spline corresponds to the highest order of the polynomials. The values *u*_0_, *u*_1_, …, *u*_*k*−1_, *u*_*k*_ where the polynomial pieces connect are called *knots*.

Frequently, for a spline of order *n*, ***S*** is required to be differentiable up to order *n* − 1, that is, to be *C*^*n*−1^ at knots and *C*^∞^ everywhere else. However, it is also possible to reduce its differentiability to take into account discontinuities.

In general, any spline function ***S***(*u*) of order *n* with knots *u*_0_, …, *u*_*k*_ can be expressed as

(6) $$\begin{array}{l} S\left(u\right)=\overset{k+n+1}{\underset{j\;=\;1}{\sum }}{\text{p}}_{j}s_{j}\left(u\right), \end{array}$$

where the coefficients **p**_*j*_ are interpreted geometrically as the coordinates of the *control points* that determine the shape of the spline, and

(7) $$\begin{array}{l} {\;s}_{j}\left(u\right)={\left(u-u_{j}\right)}^{n}\text{ for}j=1,\ldots ,k,\\ \begin{array}{l} s_{k+j}\left(u\right)=u^{j-1}\text{ for}j=1,\ldots ,\left(n+1\right) \end{array} \end{array}$$

constitute a basis for the space of all spline functions with knots *u*_0_, …, *u*_*k*_, called the *power basis*. This space of functions is a (*k* + (*n* + 1))-dimensional linear space. By using other bases, a large family of spline variations is generated (Gibson and Mirtich, 1997); the most important ones are the following.

**Figure 4:**
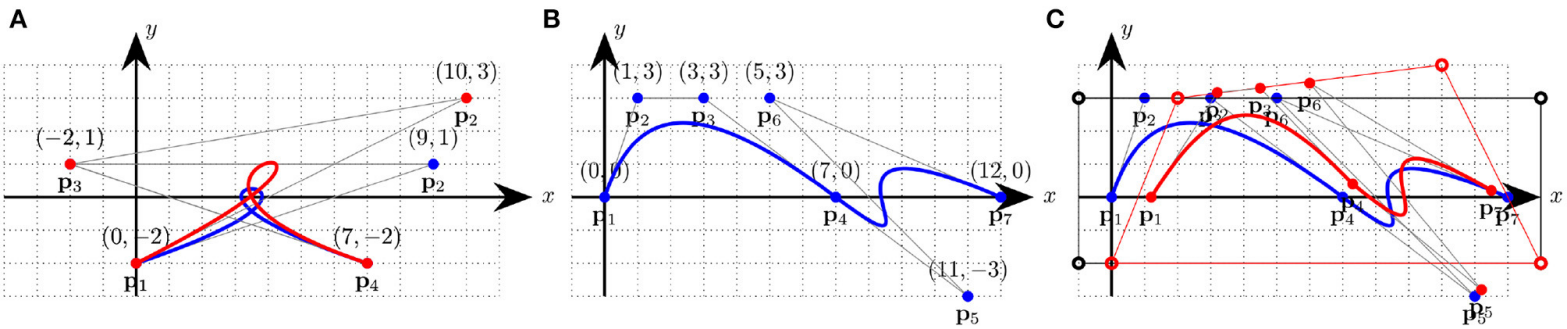
**(A)** Two Bezier curves with control points; displacing one control point (e.g., **p**_2_) affects the entire curve. In this case, the line that joins the control points **p**_1_ and **p**_2_ is the tangent of the polynomial at **p**_1_; and the line that joins **p**_3_ and **p**_4_ is the tangent at **p**_4_. **(B)** Two enchained Bezier curves; by placing **p**_3_, **p**_4_, and **p**_5_ on a line it is possible to make the curve *C*^1^ at **p**_4_. **(C)** Free-form deformation of a spline; the black circles form a lattice in a new local coordinate system, the red circles show the deformed lattice, and the red spline is the result of shifting the blue spline in accordance with this deformation.

**Bezier splines** have each segment being a Bezier curve given by
  (8) $$\begin{array}{l} \text{B}\left(u\right)=\overset{n}{\underset{i\;=\;0}{\sum }}{\text{p}}_{i}B_{i}^{n}\left(u\right), \end{array}$$
  (9) $$B_{i}^{n}(u)=\left(\begin{array}{c} n\\ i \end{array}\right){\left(1-u\right)}^{n-i}u^{i},$$
  where each **p**_*i*_ is a control point, the $B_{i}^{n}$ are the Bernstein polynomials of degree *n*, $\left(\begin{array}{c} n\\ i \end{array}\right)$ are the binomial coefficients, and *u* ∈ [0, 1]. The curve passes through its first and last control points, **p**_0_ and **p**_*n*_, and remains close to the control polygon obtained by joining all the control points, in order, with straight lines. Also, at its extremes it is tangent to the line segment defined by $\bar{{\text{p}}_{0}{\text{p}}_{1}}$ and $\bar{{\text{p}}_{n-1}{\text{p}}_{n}}$. It is easy to add and remove control points from a Bezier curve, but displacing one causes the entire curve to change, which is why usually only third-degree polynomial segments are used (see Figure 4). A two-dimensional Bezier surface is obtained as the tensor product of two Bezier curves:
  (10) $$\begin{array}{l} \text{B}\left(u,v\right)=\overset{n}{\underset{i\;=\;0}{\sum }}\overset{m}{\underset{j\;=\;0}{\sum }}B_{i}^{n}\left(u\right)B_{j}^{m}\left(v\right){\text{p}}_{i,j}. \end{array}$$
**B-splines** are more stable, since changes to the positions of control points induce only local changes around that control point, and the polynomials pass through the control points (de Boor, 1976). They are particularly suitable for 3D reconstructions. Song and Bai (2008) show how they can be used to fill holes and smooth surfaces originally captured as dense clouds of points, while producing a much more compact and manipulable representation.
**Catmull-Clark surfaces** approximate points lying on a mesh of arbitrary topology (Catmull and Clack, 1978).
**Non-uniform rational B-splines (NURBS)** are notable because they can represent circles, ellipses, spheres, and other curves which, in spite of their commonness and simplicity, cannot be represented by polynomial splines. They achieve this by introducing quotients of polynomials in the basis^2^.
**T-splines and non-uniform rational Catmull-Clarck surfaces with T-junctions (T-NURCCs)** allow for high-resolution representations of 3D deformable objects with a highly reduced number of faces and improved representations of joints between surface patches, by introducing connections with the shape of a T between edges of the shape (Sederberg et al., 2003).

Splines are a very flexible tool for representing all sorts of deformable shapes. They are extremely useful for signal and image processing (Unser, 1999) as well as for computer animation (Maraffi, 2004) and shape reconstruction of 2D and 3D deformable objects (Song and Bai, 2008; Prasad et al., 2010). *Active contours* (see section 3.4.2 and Kass et al., 1988), also known as *snakes*, are splines governed by an energy function that introduces dynamic elements to the shape representation and are used for tasks, such as object segmentation (Marcos et al., 2018; Chen et al., 2019; Hatamizadeh et al., 2019).

The advantage of splines is that compact representations of deformable objects can be built on them in accordance with the complexity of their shape at each time. When new corners or points of high curvature appear, more control points can be added for an adequate representation, and if the shape becomes simplified these points can be removed. However, this flexibility also makes splines sensitive to noise and can lead to computation of erroneous deformations. Hence, learning the dynamics of such a representation is difficult with current learning algorithms, and a general solution remains an open problem.

#### 2.2.2. Modal Decompositions

In modal decomposition, a curve or a surface is expressed as the sum of terms in a basis, whose elements correspond to frequency harmonics. The sum of the first modes constituting the surface gives a good rough approximation of its shape, which becomes more detailed as more modes are included (Montagnat et al., 2001). Among methods for modal decomposition, Fourier decomposition is in widespread use. A curve may be represented as a sum of sinusoidal terms and a surface as a combination of spherical harmonics $Y_{l}^{m}\left(\theta ,\varphi \right)$ which are explicitly parameterized:

(11) $$\begin{array}{l} S\left(r,\theta ,\varphi \right)=\overset{\infty }{\underset{l\;=\;0}{\sum }}\overset{l}{\underset{m\;=\;-l}{\sum }}c_{l}^{m}r^{l}Y_{l}^{m}\left(\theta ,\varphi \right), \end{array}$$

where *r*^*l*^ is a normalization factor for ***Y*** and the $c_{l}^{m}$ are constants. It is also possible to use other bases that may be more suitable for other shapes, such as surfaces homeomorphic to a sphere, torus, cylinder, or plane.

Modal decomposition has mostly been used in model-based segmentation and recognition of 2D and 3D medical images (Szekely et al., 1995). Its main advantage is that it creates a compact and easy-to-manipulate representation of objects whose shape can be described as a linear combination of a few dominant modes of deformation. The disadvantage of such methods is that it is easy for them to miss details in objects, such as small dents, because shapes are approximated by a limited number of terms.

### 2.3. Free-Forms

Free-form deformation is a method whereby the space in which a figure is embedded is deformed according to a set of control points of the deformation (Moore and Molloy, 2007; see Figure 4C). It can be used to deform primitives, such as planes, quadrics, parametric surface patches, or implicitly defined surfaces. The deformation can be applied either globally or locally. A local coordinate system is defined using a parallelpiped so that the coordinates inside it are **p** = {*x*_1_, *x*_2_, *x*_3_} with 0 < *x*_*i*_ < 1 for all *i*. A set of control points **p**_*ijk*_ lie on a lattice. When they are displaced from their original positions, they define a deformation of the original space with new coordinates **p**′. The new position of any coordinate is interpolated by applying a transformation formula that maps **p** into **p**′. For some transformations it is enough to estimate the new coordinates of the nodes of a mesh or control points of a spline with respect to the new positions of the control points of the deformed space, and the rest of the shape will follow them, as in Sederberg and Parry (1986), where a trivariate tensor product Bernstein polynomial was proposed as the transformation function. Loosely related are multigrid representations, which also allow for local management of deformation (Xian et al., 2019).

### 2.4. Discrete Representations

Discrete representations contain only a finite fixed number of key elements describing them, mainly points and lines. Representations that fall into this category include the following:

**Meshes** are collections of vertices connected through edges that form a graph. Common shapes for their faces are triangles (*triangulations*), quadrilaterals, and hexagons for surfaces, and tetrahedrons for volumes. A special case consists of the *simplex meshes*, which have a constant vertex connectivity. This type of shape representation permits smooth deformations in a simple and efficient manner (Delingette, 1999; Montagnat et al., 2001). Therefore, meshes are used for various tasks, such as 3D object recognition (e.g., Madi et al., 2019) and simulation of the dynamics of deformable objects (see section 3.3.1) with efficient coding (Arvanitis et al., 2019).
**Skeletons** are made of rigid edges connected by joints that allow bending. The position and deformation of elements attached to the skeleton are defined with respect to their assigned bone. Skeletons tend to be used together with the method known as *skinning*, where a deformable surface is attached to the bone and softens the visual appearance of the articulated joints through interpolation techniques. By nature skeletons are designed to model articulated deformations. Schaefer and Yuksel (2007) proposed a method to automatically extract skeletons by detecting articulated deformations.
**Deformable templates** are parameterized representations of a shape that specify key values of salient features in an image. They deform with greater ease around these features. The features can be peaks and valleys in the image intensity, edges, and the intensity itself, as well as points of high curvature^3^ (sharp turns or bends; see Yuille et al., 1992; Basri et al., 1998). Deformable templates are mainly used for object recognition and object tracking (e.g., Ravishankar et al., 2008; Xia et al., 2019; Gallardo et al., 2020). They support the particular relevance of critical points in modeling deformations, which could make them key to developing a robot's ability to generate its own optimal representation of a deformable object. The use of deformable templates is explored and illustrated by experiments on natural and artificial agents in Arriola-Rios et al. (2013).
**Landmark points** are points that would remain stable across deformations. They can be corners, T-junctions, or points of high curvature. For example, when a rectangular sponge is pushed, its corners will still be corners after the deformation, while the point of contact with the external force will become a point of high curvature during the process and will remain as such; these are all stable points. In particular, landmark points can correspond to control points of splines (Blake et al., 1998). Methods for further processing, such as the application of deformations, can work more efficiently if they focus only (or mainly) on landmark points rather than on the whole representation (Cootes and Taylor, 2004).
**Particles** are idealized zero-dimensional dots. Their positions are specified as a vector function parameterized by time, ***P***(*t*). They can store a set of attributes, such as mass, temperature, shape (for visualization purposes), age, lifetime, and so on. These attributes influence the dynamical behavior of the particles over time and are subject to change due to procedural stochastic processes. The particles can pass through three different phases during their lifetime: generation, dynamics, and death. However, manipulating them and maintaining constraints, such as boundaries between them can become non-trivial. For this reason, particles are used mainly to represent gases or visual effects in animations, where the interaction between them is very limited (Nealen et al., 2006).
**Clouds of points** are formed by large collections of coordinates that belong on a surface. They are frequently obtained from 3D scanned data and may include the color of each point. A typical problem consists in reconstructing 3D surfaces from such clouds (Newcombe and Davison, 2010; Makovetskii et al., 2020). Cretu et al. (2009) gives a comparative review of several methods for efficiently processing clouds of points and introduces the use of self-organizing neural gas networks for this purpose. Clouds of points can be structured and enriched with orientations of the 3D normals, as well as other feature descriptors for perceptual applications (Martínez et al., 2019).

## 3. Representing Dynamics for Deformable Objects

After an object's shape is defined as described in section 2, a suitable model of the dynamics can be used to register and predict deformations as a robot interacts with the object (Figures 1ii,iii). In this section, we introduce some of the most commonly used models from different fields (e.g., computer graphics; see Gibson and Mirtich, 1997; Nealen et al., 2006; Moore and Molloy, 2007; Bender et al., 2014) for predicting the dynamics of deformable objects. In robotics, the important features used to select an appropriate model are computational complexity (e.g., for real-time perception and manipulation), physical accuracy or visual plausibility, and simplicity or intuitiveness (i.e., the ability to implement simple cases easily and to be built on iteratively to accommodate more complex cases). Therefore, we divide the models into three classes: (1) particle-based models, which are usually computationally efficient and intuitive but physically not very accurate; (2) constitutive models, which are physically accurate but computationally complex and not very intuitive; and (3) approximations of constitutive models, which aim to decrease the computational complexity of constitutive models through approximations.

### 3.1. Background Knowledge of Deformation

First, we briefly review some background information about the physics and dynamics of deformation. Initially, the object is in a rest shape ***S***_0_. In the discrete case it could be $S_{0}=\left\{{\text{p}}_{i}^{0}=\left\{x_{i}^{0},y_{i}^{0},z_{i}^{0}\right\}\in {\mathbb{R}}^{n=3},i\in N\right\}$ where *N* is the number of points constituting the shape of the object. Then, when an external force **f**_ext_ acts on the object, such as gravitational force or force applied by a manipulator, the object deforms and its points move to a new position **p**^new^. In physics-based models, the resulting deformation is typically defined using a displacement vector field **u** = **p**^new^ − **p**^0^. From this displacement, the deformation can be computed through the stress σ (i.e., the force applied per area of the object shape) and the strain ϵ (i.e., the ratio of deformation to the original size of the object shape). The stress tensor σ is usually calculated for each point on the object shape using Hooke's law, **σ** = **E**ϵ, where **ϵ** can be calculated as $\epsilon =\frac{1}{2}\left(\nabla \text{u}+\nabla {\text{u}}^{T}\right)$ with ∇**u** denoting the spatial derivative of the displacement field,

(12) $$\begin{array}{l} \nabla \text{u}=\left(\begin{array}{ccc} \partial u_{x}/\partial x & \partial u_{x}/\partial y & \partial u_{x}/\partial z\\ \partial u_{y}/\partial x & \partial u_{y}/\partial y & \partial u_{y}/\partial z\\ \partial u_{z}/\partial x & \partial u_{z}/\partial y & \partial u_{y}/\partial z \end{array}\right); \end{array}$$

**E** is a tensor that is dependent on the real physical material properties of the object, such as Young's modulus *E* and Poisson's ratio υ. These properties are parameters in constitutive models (e.g., finite element models). Constitutive models describe strain-stress relationships as the response of materials (e.g., elastic or plastic) to different loads (e.g., forces applied) based on the material properties; they are commonly used to simulate deformation because of their high physical accuracy.

To simulate the dynamical behavior of deformation over time, Newton's second law of motion is employed. Let ${\text{p}}_{i}^{t}$ be the position of particle *i* at time *t*:

(13) $$\begin{array}{l} {\text{v}}_{i}^{t}={\overset{∙}{\text{p}}}_{i}^{t},\;{\text{a}}_{i}^{t}={\overset{∙}{\text{v}}}_{i}^{t},\;m_{i}{\text{a}}_{i}^{t}={{\text{f}}_{\text{ext}}}_{i}^{t}, \end{array}$$

where *m*_*i*_, ${{\text{f}}_{\text{ext}}}_{i}^{t}\in {\mathbb{R}}^{3}$, ${\text{a}}_{i}^{t}\in {\mathbb{R}}^{3}$, and **v**^*t*^ ∈ ℝ^3^ are respectively the mass, external forces, acceleration, and velocity at time *t*, and ${\overset{∙}{\text{p}}}_{i}^{t}=\frac{\left({\text{p}}_{i}^{t+\Delta t}-{\text{p}}_{i}^{t}\right)}{\Delta t}$ and ${\overset{∙}{\text{v}}}_{i}^{t}=\frac{\left({\text{v}}_{i}^{t+\Delta t}-{\text{v}}_{i}^{t}\right)}{\Delta t}$ are first-order time derivatives of the position and velocity, respectively, which are approximated using finite differences. Then, according to these derivative approximations, in each time step Δ*t* the points move according to a time integration scheme. The simplest such scheme is explicit Euler integration:

(14) $$\begin{array}{l} {\text{p}}_{i}^{t+\Delta t}={\text{p}}_{i}^{t}+{\text{v}}_{i}^{t}\Delta t, \end{array}$$

(15) $$\begin{array}{l} {\text{v}}_{i}^{t+\Delta t}={\text{v}}^{t}+\frac{1}{m}{{\text{f}}_{\text{ext}}}_{i}\Delta t, \end{array}$$

where ${\text{v}}_{i}^{t}$ and ${\text{p}}_{i}^{t}$ are the velocity and position of point *i* at time *t*. We remark that explicit Euler integration can cause problems, such as unrealistic deformation behavior (e.g., overshooting). There are other more stable integration schemes (e.g., implicit integration, Verlet, Runge-Kutta; see Hauth et al., 2003) that can be used.

Such a dynamical model can be represented simply as *G* (***S***_0_, **f**_ext_, θ), where input to the model *G* includes the initial state of points ${\text{p}}^{0}\in S_{0}$ of the object and the external forces **f**_ext_; θ represents model parameters that could be related to material properties (e.g., *E*, υ), as in constitutive models, to simulate desired deformations. Then, within *G*, the deformation is computed and the points **p**^*t*^ are iterated to time state *t* using an integration scheme, such as (14) and (15).

### 3.2. Particle-Based Models

#### 3.2.1. Particle Systems

**Figure 5:**
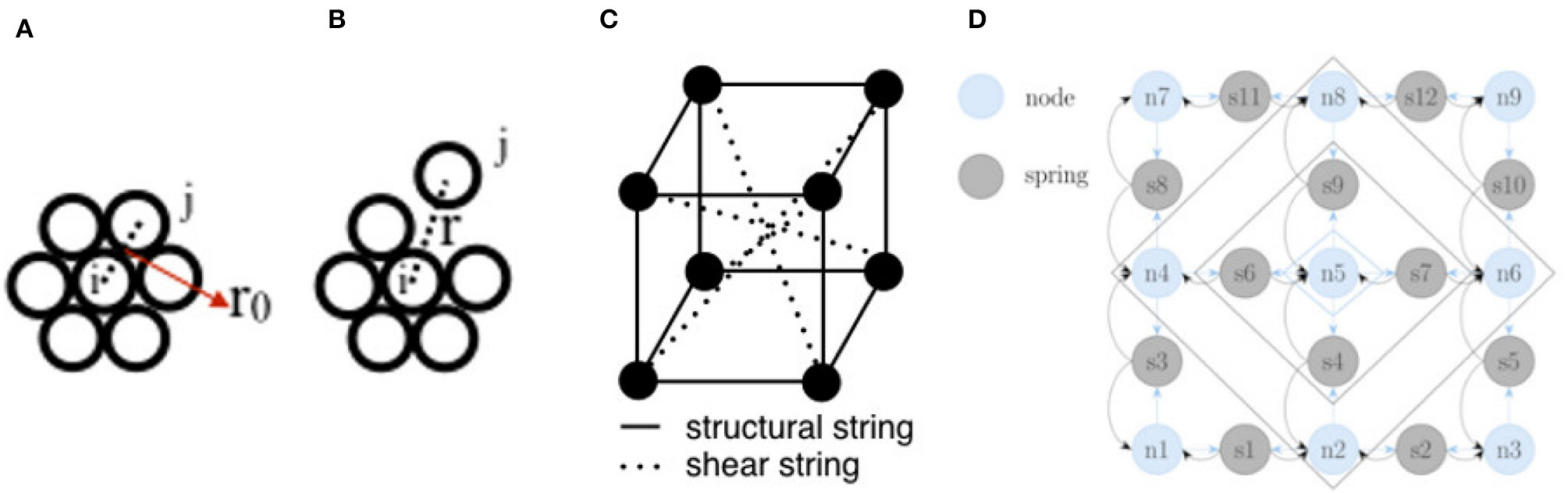
**(A)** A simple circular deformable object represented in 2D with *N* = 7 particles in the particle system; the particles are in static equilibrium (initial rest state). **(B)** When the distance *r* between the particles *i* and *j* increases, new forces exerted on *i* are calculated and the particles move. **(C)** A mass-spring model of a simple cubic deformable object with *N* = 8 particles in 3D; the particles are connected with structural and shear springs that enable the object to resist longitudinal and shear deformations. **(D)** In Nurnberger et al. (1998), the neurons of a recurrent neural network represent the mass nodes (light blue) and springs (gray) of a mass-spring model; the activation functions between the neurons of the network were devised to reproduce the mass-spring system equations.

In a particle system, a solid object shape ***S*** is represented as a collection of *N* particles (see section 2.4). These particles are initially in an equilibrium position, ${\text{p}}_{i}^{0}=\left\{x_{i}^{0},y_{i}^{0},z_{i}^{0}\right\}\in {\mathbb{R}}^{3}$, which can be regarded as the initial coordinates of each particle *i* ∈ {1, …, *N*} (Figure 5A). When an external force is applied, the object deforms and the particles move to new coordinates ${\text{p}}_{i}^{t}$ based on physics laws, in particular Newton's second law of motion (13), according to a time integration scheme, such as (14) and (15) (Figure 5B).

Although particles are usually used to model objects, such as clouds or liquids, there are also particle frameworks for simulation of solids. These frameworks are based on so-called dynamically coupled particles that represent the volume of an object (Tonnesen and Terzopoulos, 2000). The advantage of particle systems is their simplicity, which allows simulation of a huge number of particles to represent complex scenes. A disadvantage of particle systems is that the surface is not explicitly defined. Therefore, maintaining the initial shape of the deforming object is difficult, and this can be problematic for applications, such as tracking the return of elastic objects to their original shape after deformation during robotic manipulation. Hence, for objects that are supposed to maintain a given structure, particle-based models with fixed particle couplings are more appropriate, such as models that employ meshes for shape representation.

#### 3.2.2. Mass-Spring Systems

Mass-spring (MS) models use meshes for shape representation (see section 2.4). In such a model *N* particles are connected by a network of springs (Figure 5C). As in particle systems, particle motion is simulated using Newton's second law of motion (13). However, there are other forces between the connected particles, say *i* and *j*, that affect their motion, in particular the spring force ${\text{f}}_{\text{s}}\left({\text{p}}_{i}\right)=k_{\text{s}}\left(|\left({\text{p}}_{j}-{\text{p}}_{i}\right)|-l_{ij}\right)\frac{\left({\text{p}}_{j}-{\text{p}}_{i}\right)}{\left|\left({\text{p}}_{j}-{\text{p}}_{i}\right)\right|}$, where *k*_s_ is the spring's stiffness and *l*_*ij*_ is the rest length of the spring, and the damping force **f**_d_(**p**) = *k*_d_(**v**_*j*_ − **v**_*i*_) of the spring, where *k*_d_ is the damping coefficient. Then, the equation of motion (13) becomes

(16) $$\begin{array}{l} m_{i}{\text{a}}_{i}={\text{f}}_{\text{ext}}\left({\text{p}}_{i}\right)+{\text{f}}_{\text{d}}\left({\text{p}}_{i}\right)+{\text{f}}_{\text{s}}\left({\text{p}}_{i}\right). \end{array}$$

For the entire particle system, this can be expressed in matrix form as

(17) $$\begin{array}{l} \text{M}\text{a}+\text{D}\text{v}+\text{K}\text{u}={\text{f}}_{\text{ext}}, \end{array}$$

where **M** ∈ ℝ^3*N*×3*N*^, **D** ∈ ℝ^3*N*×3*N*^, and **K** ∈ ℝ^3*N*×3*N*^ are a diagonal mass matrix, a diagonal damping matrix, and a stiffness matrix for *n* = 3 dimensions. The MS system can be represented as a model *G*_MS_(***S***_0_, **f**_ext_, θ), where input to *G*_MS_ consists of the initial state of the mesh shape ${\text{p}}^{0}\in S_{0}$, the external forces **f**_ext_, and the model parameters θ = {*k*_s_, *k*_d_}, which can be changed (tuned) to determine object deformability.

MS systems are a widely used type of physics-based model for predicting and tracking object states during robotic manipulation, since they are intuitive and computationally efficient (Schulman et al., 2013). However, the spring constants are difficult to tune according to the material properties to obtain the desired deformation behavior. One way to overcome this tuning problem is to use learning algorithms and reference solutions (Bianchi et al., 2004; Morris and Salisbury, 2008; Arriola-Rios and Wyatt, 2017). Another disadvantage of MS models is that they cannot directly simulate volumetric effects, such as volume conservation in its basic formulation. To simulate such effects, Teschner et al. (2004) introduced additional energy formulations. Also, the behavior of an MS model is affected by the directions in which the springs are placed; to deal with this issue, Bourguignon and Cani (2000) added virtual springs to compensate for this effect. In addition, Xu et al. (2018) proposed a new method by introducing extra elastic forces into the traditional MS model to integrate more complex mechanical behaviors, such as viscoelasticity, non-linearity, and incompressibility.

#### 3.2.3. Neural Networks

Nurnberger et al. (1998) designed a method for controlling the dynamics of an MS model using a recurrent neural network (NN). Different types of neurons are used to represent the positions **p**, velocities **v**, and accelerations **a** of the mass points (nodes) and the springs (spring nodes) of the mesh shape ***S*** (Figure 5D). The differential equations governing the behavior of the MS system are codified in the structure of the network. The spring functions are used as activation functions for the corresponding neurons. The whole system poses the simulation as a problem of minimization of energy. The information is propagated to the neurons in stages, starting from the mass points where the applied force is greatest, and an equilibrium point must be reached to obtain the new configuration of the nodes at each time *t*. The training is carried out with gradient descent (*backpropagation*) for the NN. In addition, Zhang et al. (2019) employed a convolutional neural network (CNN) to model propagation of mechanical load using the Poisson equation rather than an MS model.

The advantage of using an NN to control deformation is the method's flexibility, such as being able to modify the network structure during simulation (e.g., by removing springs as in Nurnberger et al., 1998) and simulate large deformations efficiently (e.g., Zhang et al., 2019).

#### 3.2.4. Position-Based Dynamics

Particle systems and MS models are force-based models where, based on given forces, the velocities and positions of particles are determined by a time integration scheme. In contrast, position-based dynamics (PBD) models compute the positions directly by applying geometrical constraints in each simulation step. PBD methods can be used for various purposes, such as simulating liquids, gases, and melting or visco-elastic objects undergoing topological changes (Bender et al., 2014). Here we focus on a special PBD method, called meshless shape matching (MSM; see Müller et al., 2005), that is used to simulate volumetric solid objects while preserving their topological shape.

**Figure 6:**
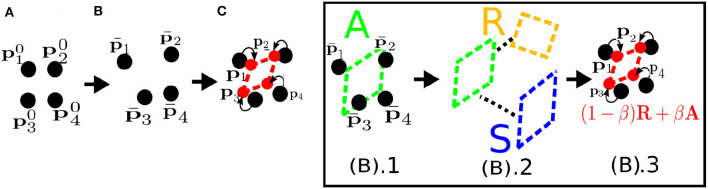
**(A)** Meshless shape matching applied to a simple object consisting of *N* = 4 particles. **(B).1** The method estimates the optimal linear transformation **A** that allows the particles to move to the actual deformed positions **p** with respect to the rest state **p**^0^, as in **(C)**. **(B).2** Then **A** is decomposed into a rotational (rigid) part **R** and a symmetric (deformation) part **S**. **(B).3** The **R** transformation is used to simulate rigid motion; to simulate deformation, **A** and **R** are combined using a parameter, β.

In MSM, as in particle systems, an object is represented by a set of *N* particles without any connectivity (Figure 6A). Since there is no connectivity information between the particles, when they are disturbed by external forces (Figure 6B) they tend to adopt a configuration that does not respect the original shape **p**^0^ of the object. We call this disturbed configuration the intermediate deformed shape, ${\bar{\text{p}}}_{i}={\text{p}}_{i}^{t-1}+{\bar{\text{v}}}_{i}^{t}\Delta t$, where ${\bar{\text{v}}}_{i}={\text{v}}_{i}^{t-1}+{\text{f}}_{\text{ext}}\frac{\Delta t}{m_{i}}$. MSM calculates an optimal linear transformation, $\text{A}=\left(\underset{i}{\sum }m_{i}{\text{r}}_{i}{\text{q}}_{i}\right){\left(\underset{i}{\sum }m_{i}{\text{q}}_{i}{\text{q}}_{i}\right)}^{-1}$, between the initial shape **p**^0^ and the intermediate deformed shape $\bar{\text{p}}$ that allows preservation of the original shape of the object; here ${\text{r}}_{i}={\bar{\text{p}}}_{i}-\bar{\text{c}}$ and ${\text{q}}_{i}={\text{p}}_{i}^{0}-{\text{c}}_{0}$, with $\text{c}=\frac{1}{\overset{N}{\underset{i}{\sum }}m_{i}}\overset{N}{\underset{i}{\sum }}m_{i}{\text{p}}_{i}$ being the center of mass of the object (Figure 6B.1–3). Then, the linear transformation **A** is separated into rotational and symmetric parts: **A** = **RS** where **R** represents rigid behavior and **S** represents deformable behavior. Hence, to simulate rigid behavior, the goal (actual) position of the particles is

(18) $$\begin{array}{l} {\text{p}}_{i}=\text{R}{\text{q}}_{i}+\text{t} \end{array}$$

where **t** = **c** is the translation of the object. If the object is deformable, then **S** is also included and the goal position is

(19) $$\begin{array}{l} {\text{p}}_{i}=\left(\text{R}\left(\left(1-\beta \right)\text{I}-\beta \text{S}\right)\right){\text{q}}_{i}+\text{t}\\ \;=\left(\left(1-\beta \right)\text{R}+\beta \text{A}\right){\text{q}}_{i}+\text{t}, \end{array}$$

where β is a control parameter that determines the degree of deformation coming from the **S** matrix. If β = 0, (19) becomes (18). If β approaches 1, then the range of deformation increases. Subsequently, using the following integration scheme, the new position and velocity at time *t* are updated:

(20) $$\begin{array}{l} {\text{p}}_{i}^{t}={\bar{\text{p}}}_{i}+\alpha \left({\text{p}}_{i}-{\bar{\text{p}}}_{i}\right), \end{array}$$

(21) $$\begin{array}{l} {\text{v}}_{i}^{t}=\left({\text{p}}_{i}^{t}-{\text{p}}_{i}^{t-1}\right)/\Delta t, \end{array}$$

where α affects the stiffness of the model (similar to the MS model) and determines the speed of convergence of the intermediate positions to the goal positions. In simplest form this model can be represented as $G_{\text{MSM}}\left(S^{0},{\text{f}}_{\text{ext}},\theta \right)$, where input to the model *G*_MSM_ consists of the initial state ***S***^0^, external forces **f**_ext_, and model parameters θ = {β, α}, which can be tuned to decide the range of deformability and stiffness of the model.

The main advantages of PBD methods are their simplicity, computational and memory-wise (i.e., not needing a mesh model) efficiency, and scalability owing to their particle-based parallel nature. Also, they are able to calculate more visually plausible deformations than MS models. Hence, they have been used in a wide range of interactive graphical applications (Tian et al., 2013; Macklin et al., 2014), particularly for modeling the deformation of human body parts (Zhu et al., 2008; Sidorov and Marshall, 2014; Romeo et al., 2020), and robotic manipulation tasks (Caccamo et al., 2016; Guler et al., 2017). A disadvantage of PBD methods is that they simulate physical deformation less accurately than constitutive models, since they are geometrically motivated.

### 3.3. Constitutive Models

To simulate more physically accurate deformations, constitutive models, which incorporate real physical material properties, are used. In this subsection, we start by introducing the most commonly used constitutive models, namely finite element models, and then briefly mention other models that simplify finite element models to increase computational efficiency.

#### 3.3.1. Finite Element Method

**Figure 7:**
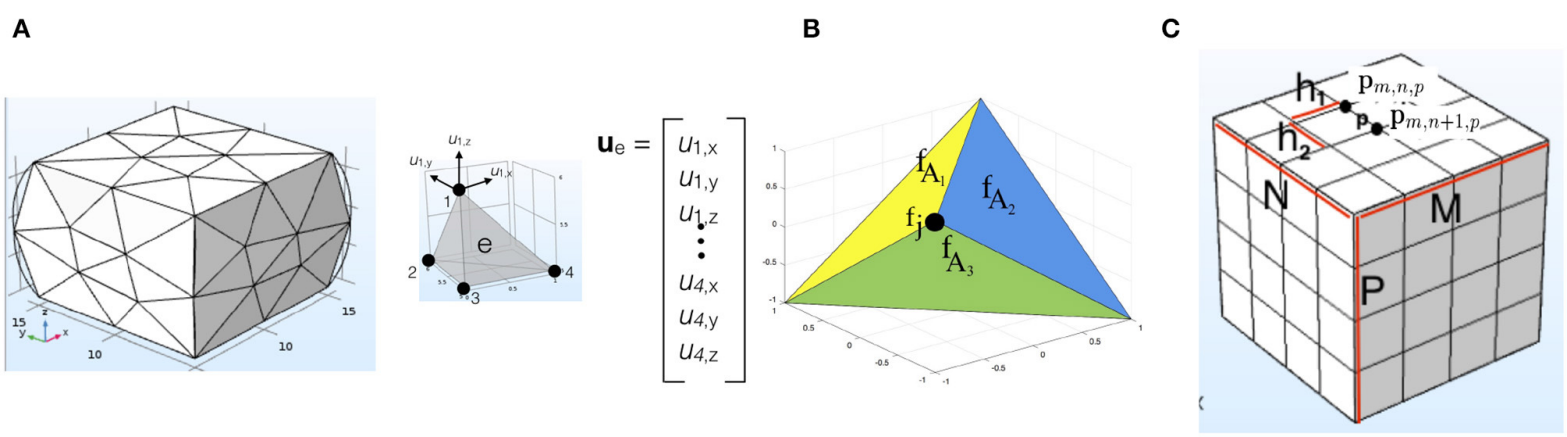
**(A)** An irregular grid as the mesh of a cubic deformed object in 3D using the finite element method (left) and an element *e* of the mesh with its *N*_*e*_ = 4 nodes (right); the arrows show the displacement fields **u**_1_ = {*u*_1,*x*_, *u*_1,*y*_, *u*_1,*z*_} at node *i* = 1, and **u**_*e*_ is the nodal displacement vector of the element *e*. **(B)** An element with node *j* and forces applied on three adjacent faces in the finite volume model. **(C)** A regular discrete mesh to be used in calculations of the finite difference method.

The finite element method (FEM) aims to approximate the true physical behavior of a deformable object by dividing its body into smaller and simpler parts called finite elements. These elements are connected through *N* nodes that make up an irregular grid mesh (Figure 7A). Thus, instead of particles, we work with node displacements. The mesh deformation is calculated through the displacement vector field **u**. For simulation, an equation of motion similar to (17) is used for an entire mesh. Usually, to decrease the computational complexity, the dynamical parts of the equation are skipped and the deformation is calculated for a static state in equilibrium (**a** = **v** = **0**). Then, the relationship between a finite element *e* and its *N*_*e*_ nodes (e.g., *N*_*e*_ = 4 for a tetrahedron as in Figure 7A) can be expressed as

(22) $$\begin{array}{l} {\text{K}}_{e}{\text{u}}_{e}={\text{f}}_{e} \end{array}$$

where ${\text{f}}_{e}\in {\mathbb{R}}^{3\times N_{e}}$ contains the *N*_*e*_ nodal forces, ${\text{u}}_{e}\in {\mathbb{R}}^{3\times N_{e}}$ is the displacement of an element between the actual and the deformed positions, and ${\text{K}}_{e}\in {\mathbb{R}}^{3N_{e}\times 3N_{e}}$ is the stiffness matrix of the element. The stiffness matrices of the different elements are assembled into a single matrix **K** ∈ ℝ^3*N*×3*N*^ for an entire mesh with *N* nodes:

(23) $$\begin{array}{l} \text{K}=\underset{e}{\sum }{\text{K}}_{e}, \end{array}$$

(24) $$\begin{array}{l} \text{K}\text{u}=\text{f}. \end{array}$$

The matrix **K** relies on the nodal displacement **u** ∈ ℝ^3 × *N*^ and the constitutive material properties (e.g., *E* and υ) to compute the nodal forces **f** ∈ ℝ^3 × *N*^ of the entire mesh. Therefore, this huge matrix **K** should be calculated at every time step *t*.

The FEM can be represented as a model $G_{\text{FEM}}\left(S^{0},{\text{f}}_{\text{ext}},\theta \right)$, which takes as input the initial state ***S***^0^, external forces **f**_ext_, and constitutive model parameters θ, such as *E* and υ. Then, within model *G*_FEM_, the new positions and velocities of the nodes are updated using a time integration scheme, such as (14) and (15).

The FEM can produce physically realistic simulations and model complex deformed configurations. Owing to these properties, FEM models have been used in many robotics applications, such as tracking (Essa et al., 1992; Petit et al., 2018; Sengupta et al., 2019) and planning manipulation around deformable objects (Frank et al., 2014). However, they can have a heavy computational burden due to re-evaluating **K** at each time step. This can be avoided by using linear FEM models where **K** in (24) stays constant^4^. The drawback is that this assumption limits the model to simulating only small deformations. Other methods that can decrease computational complexity, such as co-rotational FEM (Müller and Gross, 2004), can also be used.

#### 3.3.2. Finite Volume Method

In the finite volume method (FVM), instead of calculating the nodal forces of the mesh shape ***S*** individually as in FEM, the force per unit area with respect to a certain plane orientation is calculated. This is done by using the constitutive law for the computation of the stress tensor σ (section 3.1). Then, the total force acting on face *i* of a finite element can be calculated using the formula

(25) $$\begin{array}{l} {\text{f}}_{A_{i}}=A_{i}\sigma {\text{n}}_{i} \end{array}$$

where *A*_*i*_ is a scalar representing the area of face *i* and **n**_*i*_ is its normal vector. To calculate the nodal forces, the forces of surfaces adjacent to node *j* are summed and distributed evenly to each node (Teran et al., 2003; see Figure 7B):

(26) $$\begin{array}{l} {\text{f}}_{j}=-\frac{1}{n}\left({\text{f}}_{A_{1}}+{\text{f}}_{A_{2}}+{\text{f}}_{A_{3}}\right). \end{array}$$

The FVM model can be represented as $G_{\text{FVM}}\left(S^{0},{\text{f}}_{\text{ext}},\theta \right)$, which takes as input the initial state ***S***^0^, in which areas *A* and normals **n** can be calculated, the external forces **f**_ext_, and the constitutive model parameters *E* and υ. Then, within model *G*_FVM_, the new positions **p** of nodes of ***S***^0^ are updated at each time step *t* using a time integration scheme. Since this method is computationally more efficient than the FEM, it has been used in many computer graphics applications (Barth et al., 2018; Cardiff and Demirdžić, 2018). However, it restricts the types of deformation that can be simulated, such as the deformation of irregular meshes.

#### 3.3.3. Finite Difference Method

In the finite difference method (FDM), the volume of the object is defined as a regular *M* × *N* × *P* discrete mesh of nodes with horizontal, vertical, and stacked inter-node spacings *h*_1_, *h*_2_, and *h*_3_, respectively (Figure 7C). The nodes are indexed as [*m,n,p*] where 1 ≤ *m* ≤ *M* (parallel to the *x*-axis), 1 ≤ *n* ≤ *N* (parallel to the *y*-axis), and 1 ≤ *p* ≤ *P* (parallel to the *z*-axis), and ${\text{p}}_{m,n,p}\in {\mathbb{R}}^{3}$ is the position of the node in 3D space. The object is deformed when an external force is applied. To calculate the nodal forces, a displacement vector **u** should be calculated using spatial derivatives. This is done by defining finite difference operators between the new node positions in the deformed mesh. For example, for **p**_*m,n,p*_ the first-order finite difference operator along the *x*-axis can be defined as *d*_*x*_(**p**_*m,n,p*_) = (**p**_*m*+1,*n,p*_ − **p**_*m,n,p*_)/*h*_1_. Using the finite difference operators, the nodal forces are calculated and the deformation of the object can be computed as in the FEM (section 3.3.1).

The FDM is one of the alternative methods suggested for decreasing the computational complexity of the FEM (Terzopoulos et al., 1987). A disadvantage of this method is that it is more difficult to approximate the boundaries of objects using a regular grid for the mesh (Nealen et al., 2006), and hence the accuracy is decreased.

#### 3.3.4. Boundary Element Method

The boundary element method (BEM) computes the deformation of ***S*** by calculating the equation of motion (17) over a surface rather than over a volume as in the FEM. The boundary (surface) ***S*** is discretized into a set of *N* non-overlapping elements (e.g., mesh elements) *e*, whose node coordinates **p**_*i*_, *i* = 1, …, *N*, are the centroids of the elements. These elements represent displacements and tractions, and ***S***_*u*_ and ***S***_*r*_ are surface parts where the displacement and traction boundary conditions are defined, respectively.

The BEM provides a significant speedup compared to the FEM because it requires fewer nodes and elements. However, it only works for objects whose interior consists of homogeneous material. It has been used in the ArtDefo System (James and Pai, 1999) to simulate volumetric models in real-time. Also, it has been used to improve tracking accuracy against occlusions and spurious edges in (Greminger and Nelson, 2008).

#### 3.3.5. Long Elements Method

**Figure 8:**
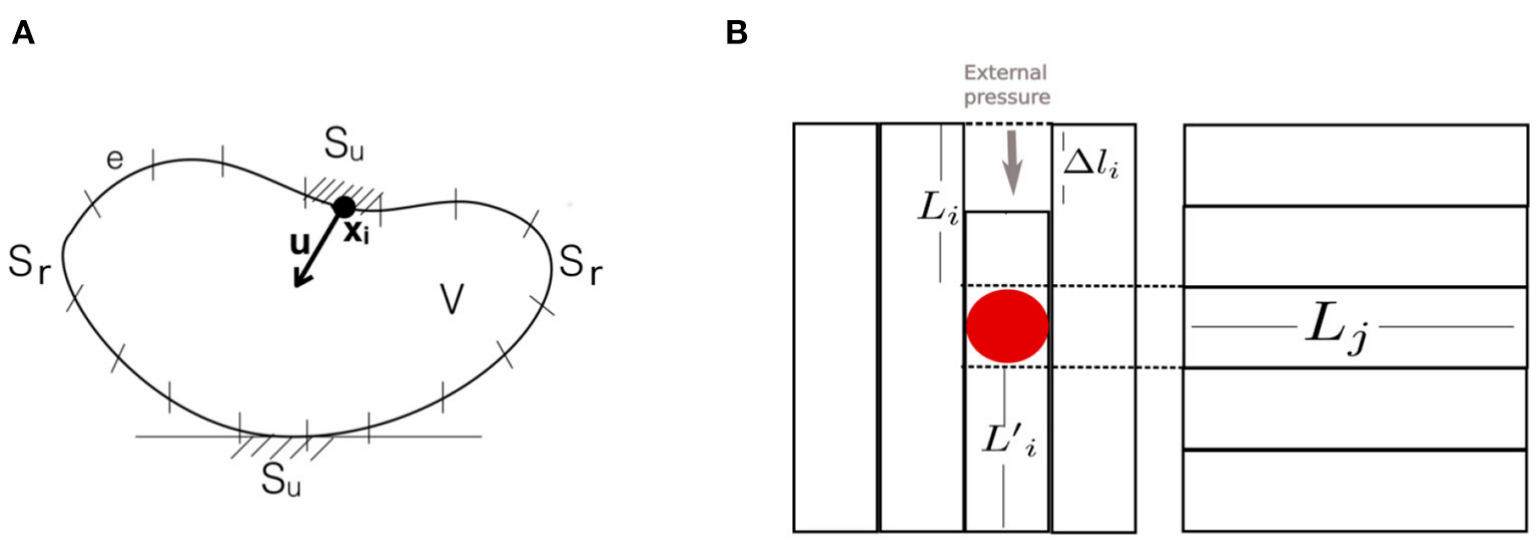
**(A)** In the boundary element method, the deformable object's shape ***S*** is discretized into a set of elements *e*; ***S*** is disturbed by a force that displaces the centroid node coordinates **p**, and the deformation is calculated based on the displacement and traction boundary conditions defined on ***S***_*u*_ and ***S***_*r*_, respectively. **(B)** A virtual object discretized as Cartesian meshes according to the long elements method. Here, for the sake of simplicity, we show a 2D object with only two axes (*i* and *j*). On the left and right figures are meshes of the object discretized with long elements *L*_*i*_ and *L*_*j*_ parallel to the *i* and *j* axes, respectively. External pressure is applied at a particular point on the surface of the object, and the resulting force *f* on the particle (red dot) is calculated using the deformations Δ*l*_*i*_ of long elements crossing the particle.

In the long elements method (LEM), a solid object is considered to be filled with incompressible fluid as in biological tissues. The volume of object shape ***S*** is discretized into Cartesian meshes (i.e., one mesh for each axis), and each mesh contains long elements (LEs) *e* ∈ {1, …, *N*_*i*_}, where *N*_*i*_ is the number of elements in the mesh shape ***S***_*i*_ discretized parallel to axis *i* (Figure 8B). The crossings of the LEs of the different axes define cells, each of which contains a particle. By calculating the state of these particles (e.g., position **p** and velocity **v**), the deformation of the object is simulated.

The state of each particle is calculated using the laws of fluid mechanics (e.g., Pascal's law). Then, a system of linear equations for each *e* that fills the object volume is created. By solving this system of equations by numerical methods, the deformation of *e*, Δ*l*_*i*_, is calculated. From Δ*l*_*i*_ the forces occurring due to deformation are computed. Here, the LE is regarded as a spring attached to a particle with known mass. As an example, in Figure 8B, pressure is applied to an object. As a result of this pressure, a force *f*_*i*_ acts on the particle along the *i*th axis and is calculated using the displacements of the crossing LEs attached to the particle:

(27) $$\begin{array}{l} f_{i}=k_{L_{i}}\left(\Delta l_{i}-\Delta l_{i^{\prime }}\right)+k_{L_{j}}\left(\Delta l_{j}-\Delta l_{j^{\prime }}\right) \end{array}$$

where *k* is the spring constant. Therefore, an LEM model can be represented as *G*_LEM_(***S***_*i*_, ***S***_*j*_, **f**_ext_, θ), which takes as input meshes of axes *i* and *j* for 2D space, external forces **f**_ext_, and model parameters, such as spring constants that determine object deformability. Subsequently, the force obtained is used to calculate the velocities and positions of the particles along each axis.

The LEM was developed for modeling soft tissues, especially for surgical simulation (Balaniuk and Salisbury, 2002). It uses a smaller number of elements than tetrahedral (e.g., FEM) and cubic (e.g., FD) meshing, so the computational complexity of the model is reduced as well. It is therefore capable of interactive real-time soft tissue simulation for haptic and graphic applications, such as robotic surgery. However, it provides only an approximation of real physical deformation and so presents a trade-off between physical accuracy and computational efficiency.

### 3.4. Approximations of Constitutive Models

#### 3.4.1. Modal Analysis

**Figure 9:**
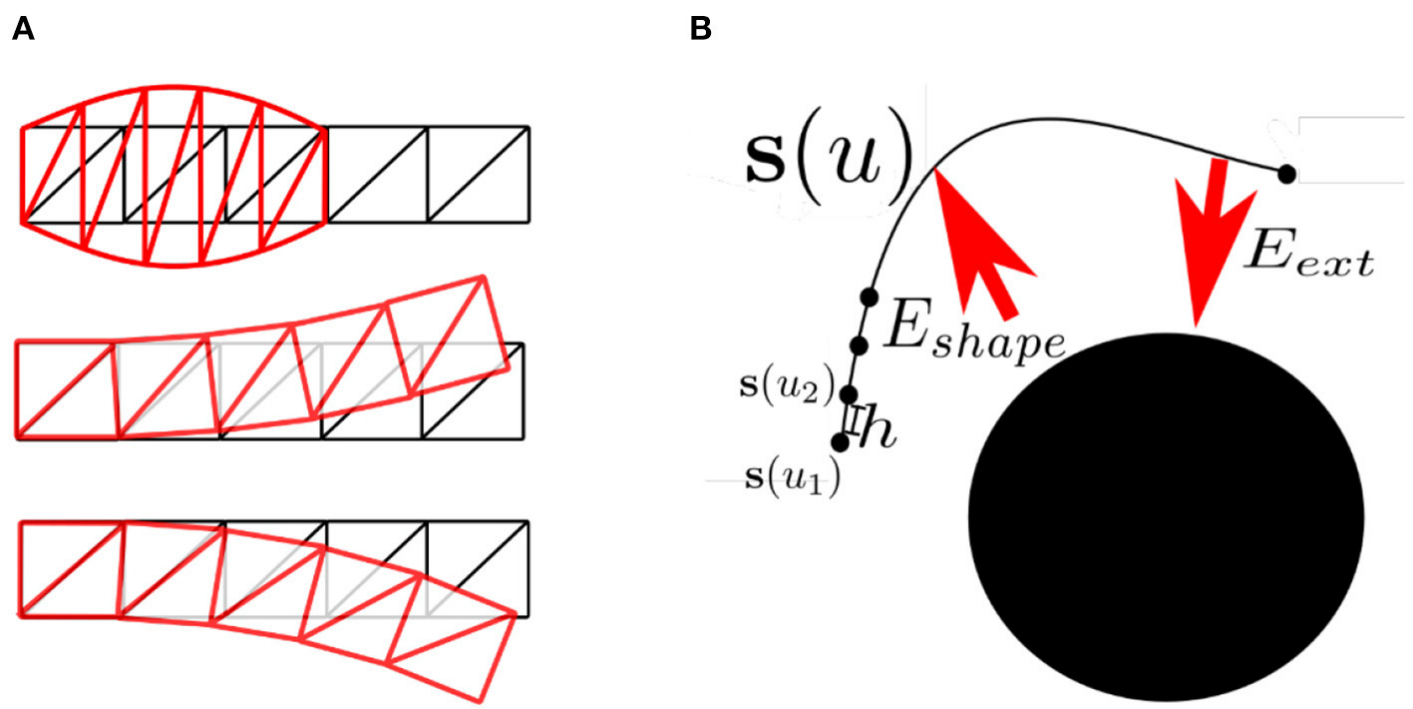
**(A)** A simple 2D rectangular object in different deformation modes in modal analysis: the upper shape is a deformation mode in response to compression, and the middle and lower shapes are deformation modes in response to bending forces from different directions. **(B)** Active contour ***S***(*u*) controlled by external (*E*_ext_) and shape (*E*_shape_) energies that attract or repel according to the shape of the object (e.g., in an image); as the object deforms, the position of ***S***(*u*) is updated iteratively.

What makes constitutive models, such as the FEM expensive is calculation of the motion with large matrices **M**, **D**, and **K** in Equation (17); for example, with *N* = 20 nodal points of a mesh shape **p** = (*x, y, z*), the calculation would involve three matrices of size 60 × 60. Pentland and Williams (1989) proposed a way of reducing this computational complexity based on a method called modal analysis. Modal analysis is used for identifying an object's vibrational modes (Figure 9A) by decoupling (17). This is done by using linear algebraic formulations (Nealen et al., 2006). Below, we outline the steps of modal analysis using these formulations, while skipping the detailed derivations.

First, the matrices are diagonalized by solving the following eigenvalue problem (i.e., *whitening transition*):

(28) $$\begin{array}{l} \text{M}\Phi \Lambda =\text{K}\Phi , \end{array}$$

where **Λ** and **Φ** are matrices containing the eigenvalues and eigenvectors of **MK**^−1^. Then, the eigenvectors of **Φ** are used to transform the displacement vector **u**:

(29) $$\begin{array}{l} \text{u}=\Phi \text{q}. \end{array}$$

By substituting (29) into (17) and multiplying by **Φ**^*T*^, the following system of equations is constructed:

(30) $$\begin{array}{l} \Phi^{T}\text{M}\Phi \ddot{\text{q}}+\Phi^{T}\text{D}\Phi \overset{∙}{\text{q}}+\Phi^{T}\text{K}\Phi \text{q}=\Phi^{T}{\text{f}}_{\text{ext}}, \end{array}$$

(31) $$\begin{array}{l} \nabla \text{M}\ddot{\text{q}}+\nabla \text{D}\overset{∙}{\text{q}}+\nabla \text{K}\text{q}={\nabla \text{f}}_{\text{ext}}, \end{array}$$

where ∇**M**, ∇**D**, and ∇**K** are all diagonal matrices. This generates 3*N* independent equations of motion for the modes:

(32) $$\begin{array}{l} {\nabla \text{M}}_{i}{\ddot{\text{q}}}_{i}+{\nabla \text{D}}_{i}{\overset{∙}{\text{q}}}_{i}+{\nabla \text{K}}_{i}{\text{q}}_{i}={{\nabla \text{f}}_{\text{ext}}}_{i}. \end{array}$$

Then, (32) can be solved analytically for **q**_*i*_ to compute the motion of each mode *i* ∈ {1, 2, …, *N*}. The matrix Φ contains a different mode shape in each of its columns (Figure 9A), i.e., Φ = [Φ_1_, Φ_2_, …, Φ_*N*_]. Hence, by analyzing the eigenvalues in **Λ**, high-frequency modes in Φ can be eliminated so that only the most dominant modes are updated using (32). This reduces the number of equations in (32) and hence lowers the computational cost significantly.

In modal analysis, as in constitutive models, taking **K** to be constant can increase the computational efficiency. However, this assumption is valid only when simulating small linear deformations and leads to errors when dynamically simulating large deformations. To overcome this problem, some methods use different formulations of the strain tensor (e.g., the Green strain as in Barbič and James, 2005) to enable simulation of larger non-linear deformations (An et al., 2008; Pan and Manocha, 2018), or adopt more data-driven approaches (e.g., by employing CNNs as in Fulton et al., 2019).

#### 3.4.2. Active Contours

Active contour (AC) models are approximations of constitutive models, such as the FEM. They were first introduced by Kass et al. (1988) in the form of the snakes model. In their simplest form they can be described as a function of a spline shape (section 2.2.1), ***S***(*u*) ∈ ℝ^*n*^ for *u* ∈ [0, 1], in an *n*-dimensional space, for example ***S***(*u*) = {**x**(*u*), **y**(*u*)} ∈ ℝ^2^ in an image *I*:ℝ^2^ → ℝ (Figure 9B). This spline is fitted to the shape of an object in the image by minimizing the following energy formulation:

(33) $$\begin{array}{l} E_{\text{snake}}=\int_{0}^{1}E_{\text{ext}}\left(S\left(u\right)\right)+E_{\text{shape}}\left(S\left(u\right)\right)du. \end{array}$$

Here *E*_ext_ depends on the contour position with respect to an attractor function *f*:

(34) $$\begin{array}{l} E_{\text{ext}}=f\left(\text{S}\left(u\right)\right), \end{array}$$

where in the *n* = 2 case the *f* (*x, y*) function could be the image intensity *I* (*x, y*), which would attract the snake to the brightest regions, or an edge detector, which would attract the snake to the edges (Moore and Molloy, 2007).

In (33), *E*_shape_ is the internal energy of the contour, which depends on the shape of the contour:

(35) $$\begin{array}{l} E_{\text{shape}}=\alpha \left(n\right)|S^{\prime }{\left(u\right)}^{2}|+\beta \left(i\right)|S^{\prime \prime }\left(u\right){|}^{2}. \end{array}$$

The first-order derivative |***S***′(*u*)^2^| controls the length of the contour, and the goal is to minimize the total length. The second-order derivative |***S***″(*u*)|^2^ controls the smoothness of the contour; this term enables the contour to resist stretching or bending by external forces due to *f* (***S***′(*u*)) and is used to regularize the contour. The weight parameters α and β determine elasticity and rigidity, respectively.

The energy *E*_snake_ can be discretized into *N* parts as **s**_*i*_(*u*) where the *u*_*i*_ = *ih*, for *i* ∈ {1, …, *N*}, are knots and $h=\frac{1}{N}$:

(36) $$\begin{array}{l} E_{\text{snake}}^{*}=\overset{N}{\underset{i\;=\;1}{\sum }}E_{\text{ext}}\left({\text{s}}_{i}\left(u\right)\right)+E_{\text{shape}}\left({\text{s}}_{i}\left(u\right)\right). \end{array}$$

Then, from the discretization, the derivatives in *E*_shape_ can be approximated using finite difference operators:

(37) $$\begin{array}{l} E_{\text{shape}}\left(i\right)=\alpha_{i}\frac{|\text{s}\left(u_{i}\right)-{\text{s}}_{i-1}\left(u\right){|}^{2}}{2h^{2}}+\beta_{i}\frac{|{\text{s}}_{i-1}\left(u\right)-2{\text{s}}_{i}\left(u\right)+{\text{s}}_{i+1}\left(u\right){|}^{2}}{2h^{4}}. \end{array}$$

To find the contour that minimizes the total energy, $E_{\text{snake}}^{*}$ is minimized. The resulting expression is then put into matrix form and used to update the position of the contour iteratively in time by using a time integration scheme as demonstrated in Kass et al. (1988). To represent an AC in 3D, some additional parameters are included in the shape energy formulation: the elasticity parameter β is defined along the third axis as well, and an extra parameter is added to control the resistance to twisting (Ahlberg, 1996).

AC models have been widely used, especially in medical imaging, for motion tracking and shape registration tasks (Williams and Shah, 1992; Leventon et al., 2000; Das and Banerjee, 2004), and they can also be combined with constitutive models to achieve greater physical accuracy (Luo and Nelson, 2001). The main disadvantage of AC models is their reliance on a good initialization of the snake contour near the desired shape in the image. To overcome this drawback, attractor functions other than image intensity, such as edge maps, have been proposed in recent years (e.g., Nisirat, 2019).

This concludes our tutorial-style description of models to provide some technical grounding in the basic mathematical approaches to deformable object modeling. The content of sections 4 (learning and estimation) and 5 (planning and control) builds on the models we have described. These sections are written in the form of broad surveys, as there is such a wide range of different approaches that we cannot cover them all in depth.

## 4. Learning and Estimation of Model Parameters

**Figure 10:**
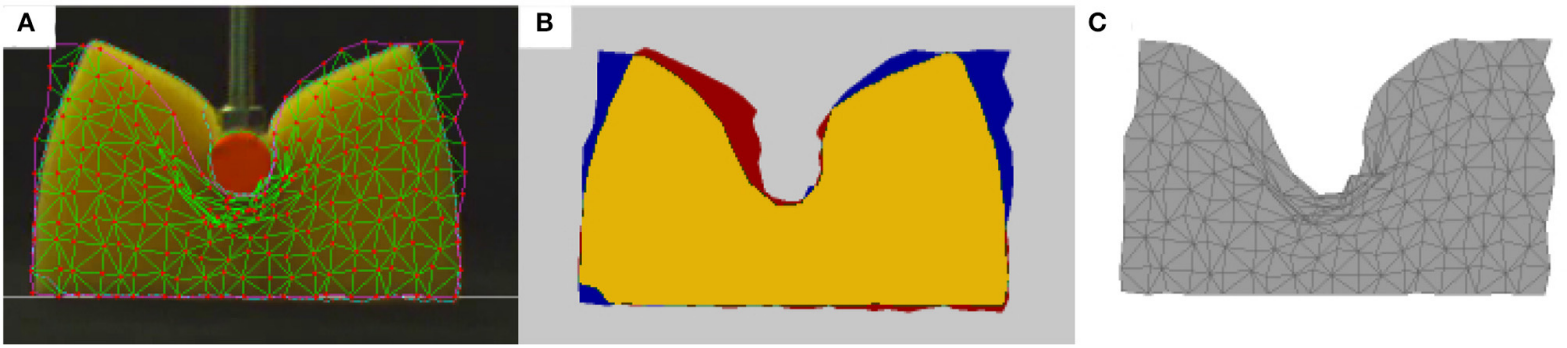
The general schema for learning: **(A)** A type of model and ground truth are selected, such as 2D images used as ground truth to calibrate a mass-spring model. **(B)** If the model's parameters have not been calibrated correctly, there will be a difference between the observed ground truth and the simulation; the observed ground truth is shown in red, the simulation in blue, and their intersection in yellow. **(C)** When the model is properly calibrated, it should be able to predict the behavior of the object if given the corresponding interaction parameters, such as external forces (e.g., contact forces of the manipulator) or geometric constraints (e.g., not crossing the floor).

In the previous sections, we introduced computational models of deformable objects that have numerous applications. However, for the models to be useful, several parameters must be known beforehand (Figure 1vi), so these models should be calibrated carefully. In this section we give an overview of some representative cases of applying various learning algorithms, which can make the calibration process autonomous (Figure 10). The methods we review can be grouped into three types of strategies: (a) estimating parameters directly, which is rarely feasible (section 4.1); (b) calibrating known physics-based models, *G*, automatically by allowing the robot to take some measurements that will help to determine the values of model parameters, θ (section 4.2.2); and (c) approximating new functions that describe the dynamics, as is done with neural networks (section 4.2.4).

### 4.1. Direct Estimation

For some models, it is possible to derive a formula to directly calculate the parameters. For example, Gelder (1998) obtained a formula for the parameter *k*_s_ of an MS model (section 3.2.2) in a static state, where the materials are non-uniform but isotropic. An isotropic material is a material whose local deformation in response to force is independent of the direction in which the force is applied. However, in a non-uniform material the response varies with the position where the force is applied. For 3D tetrahedral meshes, the spring constant *k*_s_ can be obtained from the formula

(38) $$\begin{array}{l} k_{\text{s}}=\frac{E\sum_{e}V_{e}}{|c{|}^{2}} \end{array}$$

where the sum is over the volume *V*_*e*_ of a triangular element *e* of a 3D mesh shape ***S***^0^ on its edge *c*. Young's modulus, *E*, is chosen empirically to give the desired amount of elasticity.

Direct estimation is a computationally efficient method. However, often it is not possible to do such calculations for models that rely on complex constitutive material laws as in the FEM.

### 4.2. Minimizing Error

This group of methods relies on the definition of an error function $\text{Err}\left({\text{p}}^{\theta },\hat{\text{p}}\right)=\text{dist}\left({\text{p}}^{\theta },\hat{\text{p}}\right)$ that measures the difference (e.g., Euclidean distance) between the deformation of some ground truth $\hat{\text{p}}$ and the simulated virtual deformable object position ${\text{p}}^{\theta }\in G\left(S^{0},{\text{f}}_{\text{ext}},{\text{p}}_{\text{c}},\theta \right)$, where **p**_c_ is the point of contact. The ground truth $\hat{\text{p}}$ can be obtained from camera observations of a real-world deformable object or from another, more reliable, simulation, usually an FEM simulation. Then, **p**^θ^ ∈ ***S***^θ^ is simulated with various θ values and the same interaction parameters as in the ground truth observations, such as the contact forces **f**_ext_ and positions ${\text{p}}_{\text{c}}\in S^{\theta }$ of the manipulator or geometric constraints (boundary conditions) like the object not crossing the bottom surface. The objective of the learning algorithm is to find a set of parameters θ for the model *G* that minimizes the error function $\text{Err}\left({\text{p}}^{\theta },\hat{\text{p}}\right)$ with the given interaction parameters.

Error minimization methods are usually successful at calibrating models that are difficult to tune, such as non-constitutive models (e.g., MS models) for which there is no direct link between the model parameters (e.g., *k*_s_) and the material properties (e.g., *E* and υ). However, they require the simulations to be run multiple times with different parameter values, which can be computationally expensive. Therefore, most of the time, offline estimation is performed (e.g., Leizea et al., 2017). Thus, these methods are not adaptable for online or for more dynamic deformable object manipulations. Also, in cases where the error function is not exactly convex, the algorithm may get stuck at a local minimum and result in deformations that are physically not very accurate or visually implausible.

#### 4.2.1. Exhaustive Searches

Guler et al. (2015) used exhaustive search to obtain the β value for the MSM model (section 3.2.4) $G_{\text{MSM}}\left(S^{0},{\text{f}}_{\text{ext}},{\text{p}}_{\text{c}},\beta ,\alpha \right)$. This parameter determines the degree of deformability of a material. Camera images of real-world objects are used as the ground truth, with $\hat{\text{p}}$ being the points detected on the surface of the object shape in the images. For β, uniform samples are taken from the interval [0, 1], and the deformed shape ***S***_β_ is simulated for these different β values with the corresponding interaction parameters (e.g., **f**_ext_ and **p**_c_ ∈ ***S***_β_) coming from ground truth observations. The β value that gives the minimum error between ${\text{p}}^{\beta }\in S_{\beta }$ and $\hat{\text{p}}$ is selected as the deformation parameter that represents the ground truth deformation in the best way:

(39) $$\begin{array}{l} \underset{\beta }{\text{min }}\text{Err}\left({\text{p}}^{\beta },\hat{\text{p}}\right). \end{array}$$

#### 4.2.2. Iterative Methods

Iterative methods try to decrease $\text{Err}\left(\text{p},\hat{\text{p}}\right)$ by moving the parameters step by step through the error function space. Techniques that help to ease the finding of the global minimum, such as *gradient descent* and *simulated annealing*, are frequently used to minimize the error of the model $G\left(S^{0},{\text{f}}_{\text{ext}},{\text{p}}_{\text{c}},\theta \right)$ being calibrated.

For example, Frank et al. (2014) used gradient descent to find the *E* and υ values required by an FEM model (section 3.3.1) $G_{\text{FEM}}\left(S^{0},{\text{f}}_{\text{ext}},{\text{p}}_{\text{c}},E,\upsilon \right)$. The applied forces **f**_ext_ are measured with a sensor located in the robotic manipulator; **p**_c_ is identified based on the collision point of the manipulator and the object in camera observations. Then, ***S***^0^ is deformed according to these interaction parameters. To evaluate the simulation, 3D point clouds obtained from real objects through a depth camera are used as the ground truth $\hat{\text{p}}$ and compared with the simulated FEM mesh, **p**^*E*,υ^ ∈ ***S***^*E*,υ^. Since the error function involves a computationally complex FEM model, it is difficult to compute the gradient directly. It is therefore approximated numerically by carrying out a sequence of deformation simulations that fix the interaction parameters in *G*_FEM_ and vary *E* and υ. The model thus obtained is good enough to be used for the planning of motion around deformable objects.

In contrast, Morris and Salisbury (2008) used simulated annealing to automatically calibrate an MS model and a mesh shape model based on Teschner's linear, planar, and volumetric spring energies (Teschner et al., 2004). An FEM simulation is used as the ground truth $\hat{\text{p}}$. The forces **f**_ext_ are applied at vertices of a 3D mesh ***S***^0^ that represents the object, producing deformations and resulting in a new state of static equilibrium (in which the object does not move but its shape is deformed under the influence of the forces). The objective is to reconstruct the FEM result with the MS model. To obtain the right set of parameters (e.g., *k*_s_), pools of sets of parameters are generated by randomly assigning values to the parameters of the springs. The simulations are run with these parameter sets, and those whose behavior is unstable or which do not reach static equilibrium after a certain number of steps are eliminated. For every surviving set, an error function (e.g., $\text{Err}\left({\text{p}}^{k_{\text{s}}},\hat{\text{p}}\right)$) is defined, which measures the distance between the nodes in the FEM simulation and the same nodes when displaced by MS simulation after a stable static state has been reached. Simulated annealing is used to minimize this error function.

#### 4.2.3. Genetic Algorithms

Genetic and evolutive algorithms are optimization strategies inspired by natural evolution. Combinations of elements (e.g., the spring stiffness *k*_s_ of an MS model) that constitute solutions to a defined problem (e.g., calibrating the MS model) are codified as individuals in a population. A fitness or cost function, such as $\text{Err}\left({\text{p}}^{k_{\text{s}}},\hat{\text{p}}\right)$, is defined to evaluate the potential solution represented by each individual; $\hat{\text{p}}$ may come from an FEM simulation (Bianchi et al., 2004) or from camera images of a deforming object (Arriola-Rios and Wyatt, 2017). The objective of the algorithm is to find the individuals with the highest fitness value. To accomplish this, the population is evolved by means of genetic operators, such as mutation and crossover. Mutation applies random changes to each member of the population with a certain probability (e.g., sampling new *k*_s_ values from a Gaussian distribution). Crossover creates offspring by selecting genes from a pair of individuals and combining them into a new one, also with a predefined probability (e.g., combining some *k*_s_ values sampled in the previous generation with newly sampled values in the current generation). Different criteria can be used to select which members of the population are passed onto each new generation. Although genetic algorithms do not guarantee convergence to the global optimum, local optima that are attained can still be good approximations.

#### 4.2.4. Neural Network Estimations

NNs can be used to estimate the parameters of different types of models (e.g., shape models ***S*** or dynamics models *G*). An NN is made up of several convolutional or fully connected layers. The weights of the connections between layers enable the observable parameters (e.g., the force **f**_ext_ applied by the external manipulator to deform the object) to be mapped to an output (e.g., the predicted deformed positions **p** ∈ ***S*** of points constituting the shape of the object).

In several studies, Cretu et al. used neural gas networks to segment and track the shape of deformed objects (Cretu et al., 2010, 2012; Tawbe and Cretu, 2017). The shape of the object is represented using a mesh and neural gas, which allows a more adaptable representation (e.g., by increasing the resolution of the mesh around the manipulator, which needs a higher accuracy in the deformation computation, and decreasing the resolution in the rest of the areas for computational efficiency). An NN model is used to predict the positions of the nodal points of the mesh **p** with respect to the applied force **f**_ext_. The NN is trained with data captured through force sensors and a depth camera. There are also works in which an NN is used with an FEM model to estimate parameters, such as *E* and υ and to predict the deformed positions of nodal points of the mesh of an object (e.g., Wang et al., 2018). The NN is trained offline using reference deformations with known parameters obtained from an FEM simulation and then used to predict online the deformation of real-world objects observed through a depth camera.

The advantage of such methods is that NNs provide efficient estimation similar to direct estimation, via an offline-trained NN mapping the observed input (e.g., forces applied) to an output (e.g., deformation). However, NNs require a lot of annotated training data, which may not be available or possible to obtain for every type of object that a robot might encounter.

### 4.3. Probabilistic Methods

In probabilistic approaches, usually one attempts to characterize a posterior probability, such as $p\left(\text{p}|\hat{\text{p}}\right)$ or $p\left(\text{u}|\hat{\text{p}}\right)$. The posterior probability represents the position **p** or the displacement **u** of the deformed shape with the highest probability relative to the erroneous (e.g., noisy or missing) observed deformation $\hat{\text{p}}$ coming from a sensor, such as a depth camera. Shape and dynamics models are used to represent and calculate **p** or **u**. To compute the posterior probability, methods, such as expectation maximization (Schulman et al., 2013) or sampling techniques, such as Markov chain Monte Carlo (Risholm et al., 2010) are used.

## 5. Manipulation Planning and Control

**Figure 11:**
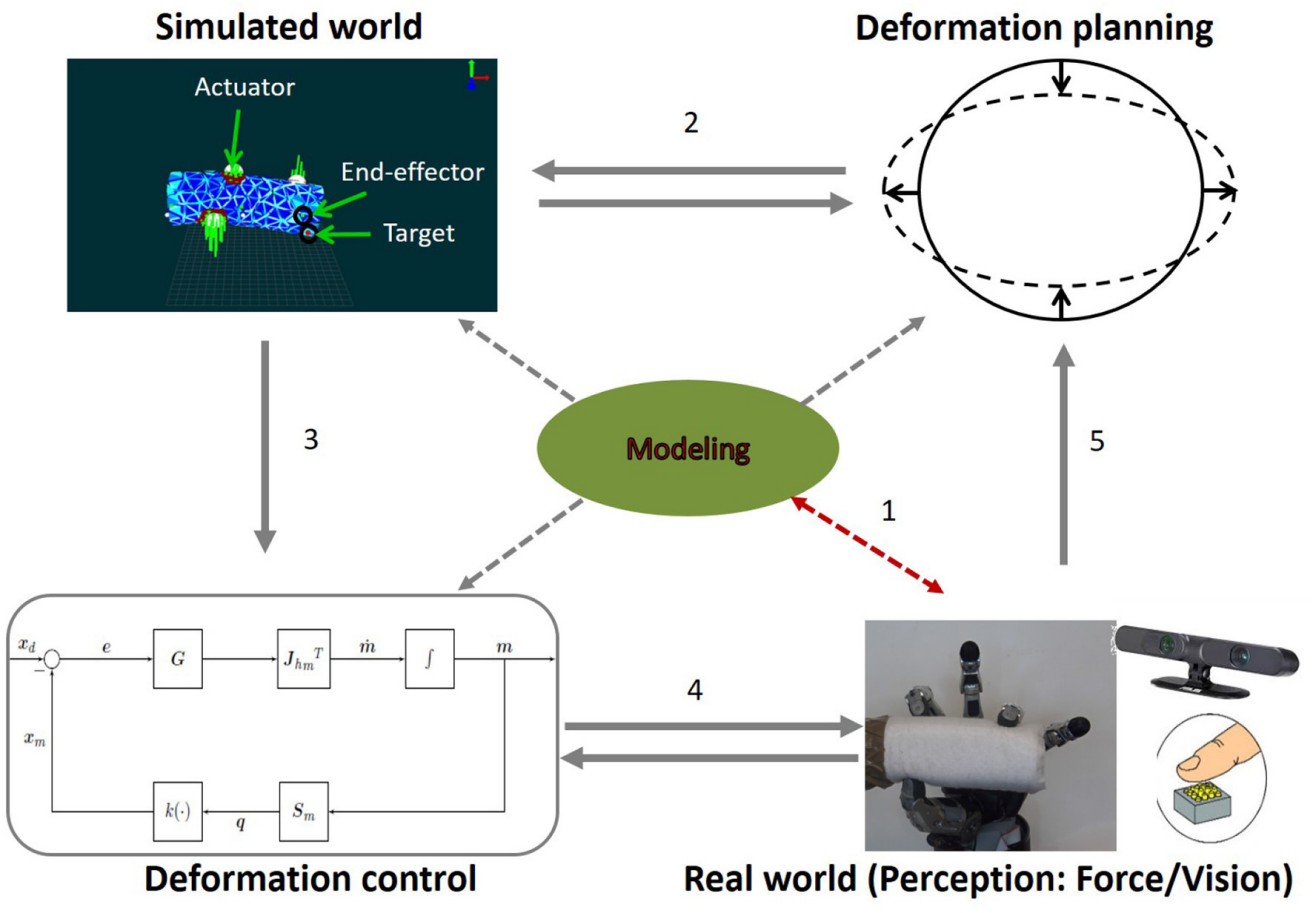
A block schema representing connections between different elements of a complex architecture for robotic manipulation of 3D deformable objects, namely modeling, perception, planning, simulation, and control. In this representation a cylindrical object and an anthropomorphic hand are taken as an example of a practical application.

Accurate tracking and prediction of deformations, supported by learning models, are needed to control the actively changing shape and motion of objects during manipulation (Figure 1iv). Active control of shape and motion consists of five main modules (Figure 11). The core is (1) the modeling of deformation (i.e., shape representation, section 2) and dynamics (section 3) and the estimation of parameters (section 4), which contributes to (2) simulation, (3) planning, and (4) control, while receiving input from (5) perception. Developing a system that can perform precise information exchanges between all these modules is still an open research problem.

Figure 11 shows an architecture for the control of deformable objects inspired by the work of Ficuciello et al. (2018), in which only some aspects of the manipulation problem are addressed. In the schema, the example of an anthropomorphic hand deforming a soft cylindrical object is considered. The manipulation is based primarily on modeling the deformation, with the model being refined and updated according to sensory perception that involves touch and vision (connection 1 in Figure 11). Then, based on the modeled shape ***S*** and dynamics *G*, planning consists in establishing the points/areas on ***S*** where concentrated/distributed external forces (called actuators) must be exerted by an end-effector to obtain desired (target) deformations. The choice of the points at which to exert the forces and the intensity of the forces are established during the planning phase, using *G* and a simulation that involves *G* and the object behavior, and this information is sent to the control (connection 3). Once the action is planned, the control module makes sure that the desired actions are correctly executed and that the expected deformations are achieved. To do this, it is necessary to consider a closed-loop control in which measurements of the state of the object and of the robot are used to correct errors (connection 4). Finally, based on the result of the action measured in the real world, a subsequent action can be planned (connection 5).

By taking into account this ideal architecture, in this section we survey the literature on robotic manipulation of deformable objects and organize the topic into two main categories, *planning of object manipulation* and *control of deformation*. The field of deformable object manipulation is still at an early stage of development. Therefore, as in Ficuciello et al. (2018), the works we review in the following cover only some of the modules depicted in Figures 1, 11 and do not describe a full system for complex deformation control of 3D objects.

### 5.1. Planning Object Manipulation

Only recently has the robotics community begun to publish results on manipulation of soft 3D objects. Most of the studies on motion planning for deformable object manipulation relate to deformable linear objects studied using mainly probabilistic roadmaps (Holleman et al., 1998; Moll and Kavraki, 2004; Saha and Isto, 2007) or deformable surgical tools and snake robots (Anshelevich et al., 2000; Bayazit et al., 2002; Teschner et al., 2004; Gayle et al., 2005). We identify two main bodies of work in the literature, namely work on deformable robots interacting with the environment and work on robots interacting with deformable environments. A few works concern planning in a complex deformable environment (Alterovitz et al., 2009; Maris et al., 2010; Patil et al., 2011).

As evidenced by the two blocks at the top of Figure 11, path planning for deformable object manipulation requires simulation of deformation, either when the deformation is the goal or when it is simply a side-effect of a planned action. In Jiménez (2012), a survey of model-based, offline planning strategies for deformable objects is presented and organized according to the type of manipulation, namely motion planning, folding/unfolding, topological modifications, and assembly. The paper is organized based on the geometric and physical characteristics of the object to be manipulated.

One of the methods adopting a model-based approach is that of Das and Sarkar (2011). In their method, three actuators are used to move the position of a point lying within the deformable object toward a goal location (target deformation). First an optimization technique, which minimizes the total force on the object to plan the location of the actuation points, is applied, and then a proportional-integral (PI) controller determines the motions of the actuators. The method uses a spline-like shape representation ***S*** (section 2.2.1) and an MS model (e.g., *G*_MS_) to simulate the dynamics of the deformable object (section 3.2.2).

Li et al. (2016) also used a model-based approach. However, rather than simulating the deformation using a dynamics model, they used a database of deformed shapes ***S*** simulated by a commercial physics simulator called Maya (Autodesk, INC., 2019). A predictive model-driven approach is used to generate a large number of instances that can be used for learning and to optimize trajectories for manipulation of deformable objects, so as to create a bridge between the simulated and real worlds. Another work that uses a database approach is Mira et al. (2015), but rather than shape models, their database contains previous grasps, which are used by their grasp planner to determine the contact points. The grasp planner was designed for flexible objects that can be grasped by exploiting their inherent flexibility. Due to similarities between the robot's hand and the human hand, the planner reproduces the action of the human hand.

A method for efficient planning of trajectories for interaction with deformable objects that is closest to the ideal schema in Figure 11 is that of Frank et al. (2014), which uses an FEM model (*G*_FEM_, section 3.3.1) and a mesh representation (section 2.4). First, the robot perceives the object through a depth camera and the model ***S*** as a mesh. Then, it calibrates the model parameters of *G*_FEM_ with the gradient descent approach by estimating the material properties (i.e., *E* and υ) through physical interaction using a force sensor (section 4.2.2). However, to avoid time-consuming computation of the FEM during online planning of manipulation, an approximate, object-specific deformation cost function is created by means of Gaussian process regression.

A few works are also available that study the problem of finding the optimal contact location for handling a deformable object. In Wakamatsu et al. (1996), the concept of bounded force-closure, which is an extension of the concept of force-closure, is introduced, and stable grasping of deformable objects based on the concept is analyzed. In Gopalakrishnan and Goldberg (2004), a new framework for holding deformable parts is developed, building on the well-established form-closure framework for holding rigid parts. Deformable parts are modeled as linearly elastic polygons with a triangular mesh in an FEM model and a given stiffness matrix. New concepts, such as D-space (deformation-space) and deform closure grasps are introduced in that work, inspired by rigid-body grasp theory.

### 5.2. Control of Deformation

Works on deformation control mostly adopt one of two approaches: model-based and real-time sensor-based.

#### 5.2.1. Model-Based Deformation Control

The methods discussed in the following require a deformation model of the target object. A simplified solution is often needed in order to derive a real-time control policy for manipulation that reduces the computational cost. In this direction, Smolen and Patriciu (2009) introduced a model-based deformation control algorithm that relies on non-linear elasticity. In their work, the boundary of a deformable object is manipulated by defining a set of control points on the object surface which must converge to desired positions. In addition, Nanayakkara et al. (2016) focus on maintaining a stable grip on a soft object. In their study a soft object, which undergoes temporal variations in its internal impedance, is presented. A control law is derived based on a relaxed stability criterion, with the aim of maintaining a stable grip on the object. The proposed controller uses only three parameters to interpret the probability of failure, which is estimated using a history of grip forces.

There are also methods that use constitutive models. In Largilliere et al. (2015), the inverse solution of an FEM model (e.g., *G*_FEM_) is used in the setting of soft robots that deform by the actions of a certain number of actuators. The goal is to generate proper actuator displacements so as to produce desired object deformations by means of desired displacements of selected control points. The desired actuator displacements are computed from the real-time inverse solution of the FEM equations, obtained by reducing the size of the constraint problem. For this purpose, a set of control points on the object is obtained using Lagrange multipliers. In Ficuciello et al. (2018), this framework is adapted to perform an active deformation control task with an anthropomorphic robot hand grasping a soft object. The method relies on *G*_FEM_ for real-time deformation control during dexterous manipulation of the 3D soft object. The goal is to generate proper forces at the fingertips during in-hand manipulation, so as to produce desired displacements of the selected control points on the object shape ***S***_2_. The desired motions of the fingers are computed in real-time as an inverse solution of *G*_FEM_, with the forces applied (e.g., **f**_ext_) by the fingertips at the contact points **p**_c_ being modeled by Lagrange multipliers (section 3.3.1). The model parameters (e.g., *E* and υ of *G*_FEM_) are initially estimated using a vision system and a force sensor. Similarly, in Lin et al. (2015) an FEM formulation, *G*_FEM_, is adopted to simulate the deformation of the object shape ***S*** based on the displacement caused by the finger contacts (e.g., **p**_c_ as the contact points and **f**_ext_ as the force applied). The simulation is used to check that the contact points do not slide while lifting the object. In this way, it is possible to properly control the motion of the fingers, so as to properly squeeze and lift the object. Moreover, in Zaidi et al. (2017), an FEM simulation based on a non-linear MS system is used to compute the deformation of an object grasped by three fingers. The contact forces are computed using the contact model based on the fingers' positions and velocities. After the contact forces are computed by simulation of the contact model guaranteeing equilibrium of the grasp, they are used as references for the grasping control strategy.

#### 5.2.2. Real-Time Sensor-Based Deformation Control

Approaches that do not use any modeling cannot predict whether the desired object shape will be achieved, but they generally have lower computational costs. Here we briefly review such approaches that do not fully conform to our ideal schema described in Figure 11 but do provide innovative and efficient control strategies for deformable objects using only sensory information. We divide them into two main categories, namely vision-based and tactile-based control strategies.

In recent work, visual servoing has been demonstrated to be a promising solution for accurate manipulation of deformation. Inspired by Smolen and Patriciu (2009), Berenson (2013) present a method of manipulating deformable objects that does not require modeling and simulation of deformation. They use a Jacobian-based method to drive the points within the deformable object toward a set of targets, assuming that they are able to sense the geometry of the object. Similar approaches are used in Cherubini et al. (2020), Hirai et al. (2001), and Wada et al. (2001). These methods align points of interest on the deformable object to targets, using a visual-servoing controller or a proportional-integral-derivative (PID) controller.

In addition, a series of works by Navarro-Alarcon et al. are based on a vision system (e.g., stereo vision) that tracks specific control points on the object and directs motion of a robotic manipulator to achieve a desired configuration (Navarro-Alarcon and Liu, 2013; Navarro-Alarcon et al., 2014; Navarro-Alarcon et al., 2016). In these papers, the deformation features based on a set of control points for describing different types of deformations are introduced for the first time. These features constitute the principal innovation of the work, together with the introduction of the *deformation Jacobian*. The deformation Jacobian defines the relationship between the manipulator's motion and the deformation feature vector, and it is estimated using Broyden's method.

In contrast, Hu et al. (2019) follows the general framework of visual servoing but combines it with a deep NN-based controller to servo-control the position and shape of a deformable object with unknown deformation properties. To describe the deformable object's deformation, a novel feature based on the fast point feature histogram (FPFH) is directly extracted from the 3D point cloud.

Apart from vision, the tactile sense is also widely used to control the shape of deformable objects during manipulation. For instance, recent work on sensor-based control by Delgado et al. uses tactile control for in-hand manipulation of elastic objects (Delgado et al., 2015; Delgado et al., 2017a,b). In their work, they adapt contact forces to different elastic properties of the object by estimating the deformability degree during the grasping process using only tactile sensors. The deformability degree can be a relative value between 0 and 1 (where 1 represents no deformation) or a classification, such as rigid body, soft elastic body, or soft plastic object, and it is related to the relationship between finger position displacement and the applied forces after the grasping process. Afterwards, contact points and forces are regulated according to the tactile information.

## 6. Discussion

Manipulating deformable objects presents more challenges than manipulating rigid objects. Developing methods to specify models that take into account the dynamics of shape change is important for robotic manipulation. In this paper we have introduced, using a combination of tutorial and review styles, some of the methods for modeling deformation. We have presented an ideal scenario for autonomous manipulation of deformable objects, which is summarized in Figure 1. According to this scenario, the problem consists of five main components.

### 6.1. Future Lines of Work

The five components of manipulating deformable objects still need improvements in the areas of shape perception, dynamics modeling, and planning/control of manipulation. Techniques from outside of robotics can help with this.

#### 6.1.1. Modeling Shape

In most state-of-the-art work, the representation of shape deformation using various models is a missing aspect that could bring in more information for controlling the shape of the object. In this direction, the different shape models described in section 2 that are not yet widely used in robotics should be explored more. For instance, the level set method, which is mostly used in medical imaging or human tracking, could be developed further to include the temporal aspect of deformation. Also, in many works, deformed object detection and segmentation from the scene are done either by hand or using simplistic color segmentation techniques (e.g., Hu et al., 2019), which may not work for complex, cluttered scenes. Hence, more autonomous methods should be investigated, such as the deformable-templates method introduced in Ravishankar et al. (2008), which is mostly used in medical imaging, or neural network-based methods that have had success in many computer vision problems, such as segmentation (Marcos et al., 2018; Chen et al., 2019; Hatamizadeh et al., 2019).

#### 6.1.2. Simultaneous Modeling, Learning, and Control

As described in sections 2 and 3, shape and dynamics models and their various parameters need to be known in order to perceive, track, and predict deformable behavior during manipulation. Currently, for realistic and accurate computation of deformation during planning and control, these models must be calibrated or learned beforehand. We have mentioned some methods for learning model parameters in section 4, but in most of the planning and control strategies (section 5), these learning techniques are applied in an offline setting because of their computational complexity. However, offline calibration may not be possible for every single object that a robot might encounter during online manipulation in real-world settings. In contrast, some control strategies do not rely on any modeling, and control of the object shape is based only on sensory measurements (e.g., Berenson, 2013), but such methods cannot predict whether the desired object shape will be achieved (section 5.2.2). To overcome this difficulty, manipulation could be controlled with a combination of model-building to incrementally learn the shape and dynamics of a previously unseen object—where the model includes the object's dynamics (e.g., force calculations and time integration scheme for updating the position and velocity)—and knowledge of properties, such as shape, mass, elasticity, etc.

In this direction, an interesting vein of work has started to emerge in recent years that we would like to mention briefly here: learning the physics of objects using deep NNs (Battaglia et al., 2016; Ajay et al., 2018). Deep NN models are similar to the NN dynamics models described in section 3.2.3 but are more scalable; for example, they can be used to model interactions between multiple objects in a single scene. We call these methods deep learning-based dynamics models. An advantage of these models is that they can learn directly from sensory observations (e.g., images of the observed scene) with high accuracy by virtue of their end-to-end differentiability, unlike the more computationally expensive analytical dynamics models in section 3, which are mostly too complex to differentiate. However, deep learning-based models have two limitations. First, most methods based on differentiable dynamics models are tested on limited scenarios with a few interacting rigid objects (e.g., balls colliding), which makes them difficult to generalize to more complicated behaviors of deformable objects. There are differentiable dynamics models that can learn more complex deformable object behavior, as in Mrowca et al. (2018) and Li et al. (2018); however, they may exhibit visually implausible deformations, such as the loss of shape preservation over time, or may not be able to deal with visual perception that includes partial or noisy observations of the state of objects, for instance due to the occlusion of a robot manipulator with an object. Furthermore, NN-based methods mostly work on entities of fixed dimensionality and do not exploit characteristics, such as the flexibility of splines.

In conclusion, for the future we need algorithms that can deal with various challenging manipulation scenarios where previously unseen objects appear in a scene (e.g., in contexts, such as cooking or nursing) by incrementally learning the shape and dynamics of a previously unseen object simultaneously while planning and controlling manipulation. To develop such methods, different approaches, such as integrating efficient differentiable models with physically accurate dynamics models, should be investigated. Also, models of dynamics should be adapted to work on different families of shape representations.

## Author Contributions

VA-R and PG were the main authors who contributed to each section. Additionally, VA-R was the main contributor to section 2, PG was the main contributor to section 3, and FF was the main contributor to section 5. JW, DK, and BS have made the substantial contributions to the conception or design of the work, revising the intellectual content critically, and providing the approval for publication.

## Conflict of Interest

The authors declare that the research was conducted in the absence of any commercial or financial relationships that could be construed as a potential conflict of interest. The handling editor declared a past co-authorship with one of the authors DK.

## Footnotes

^1^The arc length is the distance between a starting point α on the curve and the current point β. For example, the length of a 1D curve embedded in 3D space, parameterized by *u*, is given by $L=\int_{\alpha }^{\beta }\sqrt{{\dot{x}}^{2}+{\dot{y}}^{2}+{\dot{z}}^{2}}\;du$, where $\dot{x}=\frac{\partial x}{\partial u}$ and similarly for $\dot{y}$ and $\dot{z}$.

^2^https://pages.mtu.edu/~shene/COURSES/cs3621/NOTES/

^3^The curvature of a function is defined as $\kappa =\frac{d\phi }{ds}$ where ϕ is the tangential angle, with $\tan\phi =\frac{dy}{dx}$, and *s* is the arc length as defined earlier.

^4^For a detailed tutorial on how to efficiently compute the **K** matrix, we direct the reader to the notes of Müller et al. (2008).
